# Analytical Methods of Phytochemicals from the Genus *Gentiana*

**DOI:** 10.3390/molecules22122080

**Published:** 2017-11-28

**Authors:** Yan Xu, Ying Li, Katherine G. Maffucci, Linfang Huang, Rui Zeng

**Affiliations:** 1 College of Pharmacy, Southwest Minzu University, Chengdu 610041, China; Yanxu940504@163.com (Y.X.); liying@swun.cn (Y.L.); 2 Department of Chemistry, Stony Brook University, Stony Brook, NY 11794, USA; katherine.maffucci@stonybrook.edu; 3 Institute of Medicinal Plant Development, Peking Union Medical College & Chinese Academy of Medical Sciences, Beijing 100193, China; lfhuang@implad.ac.cn

**Keywords:** *Gentiana*, spectroscopy, chromatographic techniques, chemometrics, quality control

## Abstract

The genus *Gentiana* comprises approximately 400 species. Many species have a wide range of pharmacological activities and have been used therapeutically for thousands of years. To provide comprehensive guidance, utilization and quality control of *Gentiana* species, this review presents updated information concerning the recent application and progress of chemical analysis including phytochemical analysis, sample preparation and chemometrics. Detailed and comprehensive data including number of analytes, extraction/separation methods, analytical techniques and chemometrics are shown as corresponding tables. These data illustrate that the development of newly discovered compounds and therapeutic uses, understanding of the structure—activity relationship and establishment of harmonious and effective medicinal herb standards are the direction of advancement in future research.

## 1. Introduction

The genus *Gentiana* was named by Tournefort in 1700 [1]. *Gentiana*, a major group of Gentianaceae, is found throughout Europe, Asia, Northern Australia, New Zealand, North America, as well as along the Andes to Cape Horn and North Africa [2,3,4]. In China, there are 247 plant species in this genus [5]. In Chinese Pharmacopoeia (ChP) [6], the roots of *Gentiana dahurica*, *G*. *macrophylla*, *G*. *straminea*, and *G*. *crassicaulis* are prescribed as the original sources of Gentianae Macrophyllae Radix (Chinese names: “Qingjiao”). *G*. *scabra*, *G*. *triflora*, *G*. *rigescens* and *G*. *manshurica* are recorded as the raw materials of *Gentianae radix* et Rhizoma (Chinese names: “Longdan”). *Gentianae rhodanthae* Herba (Chinese names: “Honghualongdan”) is the whole plant of *G. rhodantha*. Moreover, the origin of *Gentianae radix* is the root and rihizome of *G*. *lutea* (Chinese names: “Yellow gentian”), and the root and rhizome of *G*. *scabra*, *G*. *triflora*, and *G. manshurica* are recorded as Japanese Gentianae (JP) [7]. The dried roots and rhizomes of *G*. *kurroo* are official in Indian pharmaceutical codex and substitute for *G*. *lutea* [8].

The *Gentiana* genus has important medicinal and industrial value because it is often used by the pharmaceutical and food industries in the production of alcoholic bitter beverages, food products, and traditional medicine to stimulate the appetite and improve digestion. In addition, *G*. *asclepiadea* also has ornamental value [9,10,11,12,13]. The most popular spices and herbs include “Qingjiao”, “Longdan”, “Yellow gentian”, “Honghualongdan” [14,15,16,17]. *G*. *kurroo* [18], *G*. *aristata* [19], *G*. *rhodantha* [20], and *G*. *olivieri* [21,22] have received a lot of attention. In Traditional Chinese Medicine (TCM), “Qingjiao” is an important rheumatic drug, while “Longdan” is used as a potential hepatoprotective *agent* [23]. *G*. *macrophylla*, *G*. *triflora*, *G*. *algida*, *G*. *lutea*, *G*. *olivieri*, *G*. *decumbens*, *G*. *asclepiadea* and *G*. *kurroo*, also are folk medicine to remedy digestive illnesses—most roots and rhizomes of *Gentiana* species are significant sources for the treatment of jaundice, pneumonia, constipation, pain, cough and fever [8,24,25,26,27,28,29,30]. Both in vitro and in vivo studies exhibit a variety of bioactivities including anti-oxidative and anti-inflammatory activities [31,32] as well as hypoglycemic [21], hypotensive [26], immunological [22,33], and antiproliferative [34] effects. Their high value has lead to an increase in market demand. Heavy exploitation of these species for medicinal or non-medicinal purposes threatens their existence. At present, “Qinjiao” and “Longdan”, have third-class protection and are on the List of National Key Protected Wild Herbs in China. *G*. *lutea* is listed as a rare, overexploited, or endangered species [35]. However, this demands also brings to light the need for artificial cultivation of *G. kurroo* [8,36] and *G*. *lutea* [37].

A chemical analysis of Gentians is necessary to effectively assess the differences between species and to develop long-term sustainability policies for *Gentiana* resources. Quality control through various analytical tools to guarantee purity and allow for individual assessment of the medicinal components associated with different *Gentiana* species is imperative to determine the efficacy of traditional applications. Compounds have been isolated from *Gentiana* species including essential oils, iridoids and secoiridoids, xanthones, triterpenoids, flavones. Iridoids and secoiridoids, comprising the majority of the compounds isolated, have been considered the most important substances responsible for the bioactivities [38,39]. Gentiopicroside and loganic acid, (iridoids and secoiridoid, respectively), are listed in ChP as the standard for quality control. The contents of these biological substances are affected by species, origin, growing environment, processing, storage conditions and age. Furthermore, the same plant has disparate application in different countries and areas. For instance, the root of *G. dahurica* is a common Tibetan medicine used for fighting rheumatoid arthritis (RA), but the flowers have been used as a Mongolian medicine for curing sore throat, cough and cleaning “lung-heat” [31]. Tibetan medicine utilizes the flowers of *G. straminea*, commonly known as ‘‘JIEJIGABAO’’, and Mongolian medicine utilizes the flowers of *G. macrophylla*, commonly known as “HARE-JILEZHE” [40]. The reason for this common use may be the diversity of metabolic substances. Thus, a comprehensive chemical analysis is essential for guaranteeing the efficacy and safety in clinical use.

Until now, thin-layer chromatography (TLC), nuclear magnetic resonance (NMR), atomic absorption spectroscopy (AAS), gas chromatography (GC), high-performance liquid chromatography (HPLC), capillary electrophoresis (CE) and associated techniques, like gas chromatography-mass spectrometer (GC-MS), liquid chromatography-mass spectrometry (LC-MS), liquid chromatography-tandem mass spectrometry (LC-MS-MS), have been used to investigate the metabolic substances. Some literature reviews of phytochemistry, pharmacological activities [1,5,32,39,41] and cultivation trials [37] have been published. However, advances in analytical methods have not been reported in the literature. This review is intended to bridge this gap and to trace the process analysis method of *Gentiana* species through compiled available data from the scientific literature. We wish to present scientific guidance for quality control and provide a direction for further development and utilization of the genus *Gentiana*.

In order to conduct a comprehensive literature review, we analyzed the published phytochemical and analytical techniques of *Gentiana* species to date using Science Direct (http://www.sciencedirect.com) and Pubmed (www.ncbi.nlm.nih.gov/pubmed), as well as Google Scholar (https://scholar.google.com/) and CNKI (http://www.cnki.net/). The species scientific names were checked against the plantlist databases (http://www.theplantlist.org/). The authentication of plant material used for studies is listed in the supplementary material (Table S1). The reference manager EndNote X7.7.1 was used. The software ChemDraw 15.0.0 and PubChem databases (https://pubchem.ncbi.nlm.nih.gov/) were used for graphing and identification of the chemical structures.

## 2. Phytochemical Analysis 

**Figure 1:**
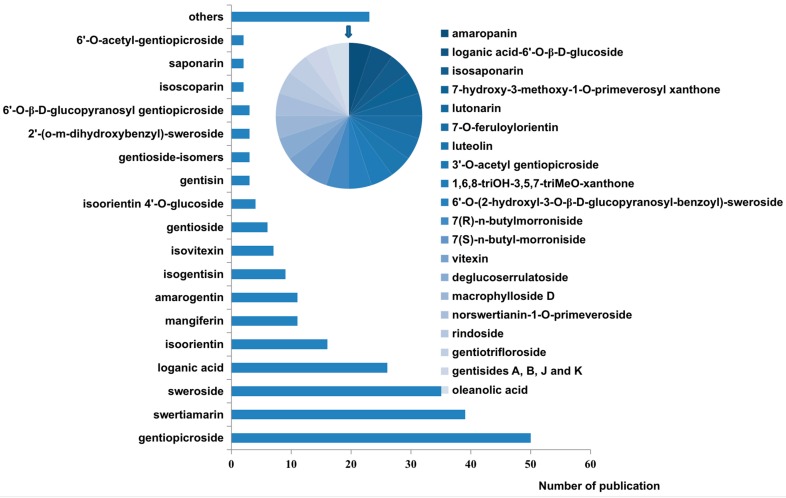
All compounds detected by analytical methods.

**Figure 2:**
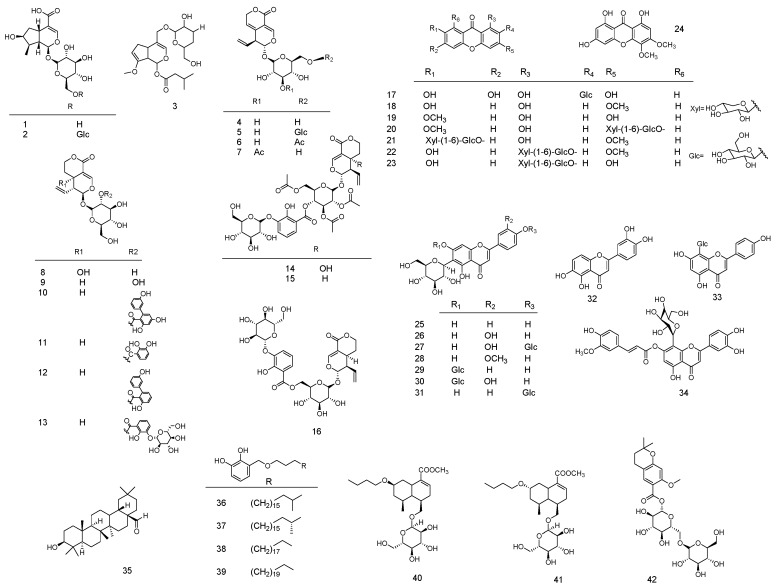
The structure of all compounds that were detected.

As of now, 593 compounds have been found in *Gentiana* species—this genus possesses an abundance of diverse compounds. Reviews on the phytochemistry of *Gentiana* have been published. Structures, species, plant parts and biological activities of compounds have been comprehensively summarized [1,39]. On this basis, we collected and summarized the data on chemical constituents and analytical chemistry, focusing on the chemical composition that is used as a marker. Figure 1 and Table 1 show all compounds which were detected through analytical techniques. Simultaneously, as shown in Figure 2, the structures of all analytes are described. Based on the new concept of quality markers, proposed by Liu et al. [42,43], we suggest that these compounds be regarded as representative ingredients for quality evaluation of *Gentiana* species. Among them, iridoids and secoiridoids (compounds **1**, **4** and **8**–**11**) are major constituents of the *Gentiana* species. Iridoids have an iridoid alcohol as their core chemical structure, forming secoiridoids via C7-C8 bond breaking. These compounds, having a hemiacetal structure, are highly unstable, providing the basis for glycoside formation in species. Xanthones (compounds **17**–**21**) contain a chromone core bearing benzene rings at C2-C3. Flavonoids (compounds **25**–**29**) are mainly glycosylated flavones. These compounds are easily detected because they contain a double bond, benzene ring, and hydroxyl group, and consequently can be visualized under UV light. At present, these chemical compounds have been detected by analytical techniques.

### 2.1. Iridoids and Secoiridoids

Although the composition of non-volatile compounds from the *Gentiana* species is relatively variable, Iridoids and secoiridoids are most abundant classes. Compounds **1**, **4**, **8**, **9**, conforming to the characteristic constituents of *Gentiana* species, were the most frequently detected [38]. The contents of compounds **4**, **9**, **8**, **1** were 1.560–0.091%, 0.814–0.003%, 0.155–0.008%, 0.257–0.022%, respectively, in *G*. *straminea*, *G*. *dahurica*, *G*. *crassicaulis*, *G*. *macrophylla*, *G*. *officinalis*, *G*. *waltonii*, and *G*. *ihassica*, which grow at high altitudes (from 2100 to 4500 m). For the first six species, in another study, these contents were quite different (10.92–2.98%, 0.21–0.001%, 1.21–0.39%, 2.15–0.16%, respectively). The main difference was reflected in the contents of compounds **4**, **8**, **1**. Moreover, the highest level of the total content of compounds **1**, **4**, **8**, **9** was observed in *G. macrophylla*. It is inferred that environmental or hereditary factors affect the accumulation of metabolites [44,45]. For compound **4**, a higher content (13%) could be observed in *G. rigescens* [46]. In roots, stems and leaves of *G. stramine*, the contents were 13.30%, 2.95% and 2.24%, respectively [61]. Component **4** accumulated mainly in the root. The result was confirmed in *G. scabra* [89]. 

Ten iridoids and secoiridoids including compounds **1**, **4**, **8**, **9** and derivatives (**5**, **16**, **11**, **6**), as well as a pair of isomers (40, 41), were simultaneously detected in *G*. *straminea*, *G*. *macrophylla*, and *G*. *crassicaulis*. Compounds **1**, **5**, **4**, **3,** relative to the proportion of the ten iridoids and secoiridoids, decreased sequentially (2.40–5.66%, 0.95–2.49%, 0.33–1.12%, 0.04–0.36%, respectively). Other compounds were present only in small amounts [47]. These data indicate that compound **4** is the most dominant in *Gentiana* species. Cultivated, wild and commercial *G. lutea* were also studied. Quantitative analysis showed the different concentrations of compounds **4**, **9**, **8**, **1**, **10** between cultivated and wild samples. The respective contents were as follows: 1.85–3.97%, 0.05–0.35%, 0.08–0.30%, 0.11–1.30%, 0.01–0.07% [11]. In other species, such as *G. decumbens*, *G. triflora*, the highest concentration was **2** (5.14–6.68%) [30].

### 2.2. Xanthones

Compared to compounds **4**, **9**, **8**, **1**, compounds **19**, **18**, **20** and **21** accounted for a lower proportion in *G. lutea* (0.03–0.48%, 0.03–0.07%, 0.03–0.43% and 0.03–0.32) [11,48,90]. In *G. rhodantha*, *G. triflora*, *G. farreri*, *G. algida*, *G. decumbens*, *G. macrophylla*, *G. rigescens*, *G. scabra*, *G. lawrencei*; *G. crassicaulis*; *G. officinalis*, *G. straminea*, Compound **5** was only detected in the first three species, and it also was the main component in *G. rhodantha* (1.90–2.19%) [30,49,50].

### 2.3. Flavonoids

In *G. triflora*, compound **27** (2.27–4.03%) had a higher concentration compared to compounds **25**, **26**, **28** and **31**. *G. algida* contained the highest level of compound **26** (2.12–3.95%). Compounds **29** and **25** reached a concentration of 0.02–0.41% and 0.14–0.71 %, respectively, in *G. triflora*, *G. macrophylla*, *G. algida*, *G. decumbens* [30].

### 2.4. Triterpenoids

At present, only ferulic acid and **35** have been analyzed by HPLC and UPLC [49,87], and **35** was discovered in the aerial parts of *G. rhodantha* and *G. farreri*. Compared to the aerial part (0.075%), a higher concentration was measured in the flower (0.182%) [87]. 

### 2.5. Other Compounds

Gentisides A, B, J, K with an alkyl 2,3-dihydroxybenzoate nucleus possessing varying alkyl chain lengths and different termini on the alkyl chains are potent inducers of neurite outgrowth on PC12 cell. By comparison with seeds, stems and leaves of *G. rigescens*, gentisides A, B, J, K mainly exist in the roots; the reported contents are 0.028%, 0.036%, 0.065%, and 0.027%, respectively. Among them, gentiside B was the major compound but was not detected in seeds. Gentisides J and K occupied a very low proportion in stems and leaves [91]. Polysaccharides have been described in *G. scabra* and *G. rigescens*. According to the literature, the different species or the same species with different extraction, purification process and analytical methods have different monosaccharides constituents and molecular weight [92,93,94,95]. Elements were investigated in *G. lutea*, *G. macrophylla*, *G. rigescens* [96,97,98,99]. The content of elements are based on the herbs own metal uptake and accumulation behaviour, and influenced by the geographical environment (soil PH). Free fatty acids as nutritious substances have recently been qualitatively and quantitativey detected in *G. straminea* and *G. dahurica*. C16, C24, C26, and C28 fatty acids showed a high content. There were more long-chain fatty acids with an even number of carbon atoms compared with an odd number of carbon atoms. Proline showed a higher level in *G. dahurica*. The effect of geographical region on the contents of fatty acids was obvious [100,101]. In bioactivity studies, simple colorimetric assays were used for the determination of the total content of certain metabolite classes, such as phenolics (Folin–Ciocalteu method), flavonoid (AlCl_3_), hydroxycinnamic acids (Arnow reagent), condensed tannins (the precipitation of proanthocyanidins with formaldehyde), and gallotannins (the reaction of potassium iodate with galloyl esters) [4,102,103,104,105]. 

### 2.6. Influence Factors

The contents of secoiridoids, xanthones, flavones, carbohydrates, and free amino acids depend on the time and year of harvest, drying process and plant organs. The period before flowering is important. The accumulation of flavonoids occurs in this period and then reaches the maximum value during flowering. For example, *G. lutea* had a higher yield of compounds **17**, **25** in the period of flowering (June and July), whereas Compound **19**, isogentisin-primeveroside, **18** and other xanthones accumulated mainly in May and April (before flowering), while a large increase occurred during flowering. Compound **10** was present during growth and the period of flowering, but was presente in minimal quantities at other times. The vegetation period (October) is another period of metabolic accumulation for Iridoids and secoiridoids. For example, compounds **4**, **8** reached the maximum in this period. The concentration increased during growth, decreased after flowering until ripening of the fruits and increased again at the appearance of seeds and onset of the quiescent period. Free amino acids had a high content during sprouting time and the quiescent period. Other metabolites, fructose, glucose and sucrose had a relatively high content during the growing period and decreased during flowering, then increased again, reaching high values in September. Gentiobiose had a high content compared to maltose and changed similar to fructose, glucose and sucrose. Trisaccharides were present in high quantity in roots except during flowering, in which there was a lower content. From these descriptions, it can be observed that there is a common feature, i.e., the concentration of these metabolites decrease in the flowering period. The drying process also affects the content. In dried roots, the contents of fructose, glucose, maltose and gentiobiose showed a higher value, whereas sucrose and gentianose showed a slightly lower value compared to fresh root. At the same time, the quantity of free amino acids in fresh roots was extremely low. This leads one to suppose that during drying, enzymatic hydrolysis and hydrolysis take place [16,106]. The effect of the drying mode on iridoids and xanthones was studied [51]. Artificial drying (40 °C) can preserve no more than 25% of compound 1 and total iridoids compared to natural dryin*G.* However, compared to fresh roots, the drying process can cause losses of compounds. The contents of compounds **1**–**4** were also affected by harvest years. A study on *G. macrophylla* at different ages reported that between two and three years, the content difference was not obvious, but in year four, the contents decreased significantly [52]. In addition, the contents of different plant parts differ. Compounds **4**, **8**, **10**, **17**, **19**, **25** and **20** in the roots were different in comparison to leaves. The contents of leaves were generally greater than those of the flowers except for compounds **4** and **19** [16,106]. 

Different growing stages show a different metabolic profile. In *G*. *rigescens*, the distribution and accumulation of metabolites during the growing stage were investigated. During plant growth, *O*-glucosyl-dihydroxy benzoyl acid gradually decreased with an increase in dihydroxy benzoyl iridoid glycosides. For example, *O*-glycosidic derivatives of compounds **21** and **25** were only detected in mature plants. It can be assumed that a complex molecular structure tends to occur in mature plants compared with proliferation stages. For compound **4**, a 1.8-fold higher concentration was observed in sample of hair root culture than in plants grown in greenhouse. In addition, this content in the root increased with decreases in the leaf and stem, when the root start to regenerate. During this stage, the growth rate was also far higher than in the leaf and stem with the occurrence of gentiopicroside growth. Thus, when roots start to regenerate, compound **4** in aerial parts may translocate into roots or transform into other metabolites. In the leaf, a negative correlation was observed between compounds **21** and **25**. Moreover, compound **21** first increased during the proliferation stage and then decreased [107]. These accumulation phenomena can be used for industrial extraction and to infer biosynthesis, transformation and degradation.

### 2.7. Essential Oils and Influential Factors 

Essential oils may be used in medical and plant pathology. They are obtained from underground and aerial parts, either all together or separated into leaves, flowers and roots [29,88,108,109]. Wild, cultivated, commercial, and different parts of *Gentiana* species contain different proportions of essential oil. This leads one to suppose that environmental conditions or methods of cultivation can affect the accumulation of volatile oils. Essential oils mainly represent benzenoids and phenylpropanoid, fatty acid derivatives, terpenoids, and sulphur-containing compounds [110]. In the genus *Gentiana*, two main classes of volatile compounds are benzenoids and terpenoids. In the study of roots of *G. lutea*, flowers of *G. triflora*, *G. scabra* and a new hybrid (*G. triflora* × *G. pneumonante*), benzenoids and derivatives reached the highest proportion (56.7%) in the new hybrid, and the lowest proportion was 11.6% in *G. triflora*. The proportions of oxygenated monoterpenes were 1.03–8.5%, 0.28–3.59%, 1.02–1.14%, 27.4–52.5%, 6.5%, respectively, in commercial, wild and cultivated samples of *G. lutea*, flowers of *G. scabra* and a new hybrid (*G. scabra* × *G. pneumonanthe*). However, monoterpenes and sesquiterpenes were not detected in most species [111]. On the contrary, flowers of *G. lutea* comprise more straight chain aliphatic hydrocarbons (4%) and 1,3-dimethoxy-3-methylbutane (3.3%). Branched saturated aliphatic hydrocarbons and alkylated benzenes possess low concentrations. Unlike flowers, straight chain aliphatic hydrocarbons and 1,3-dimethoxy-3-methylbutane were not detected in leaves [108]. In another *Gentiana* species, *G. kurroo*, sulphur-containing compounds, oxygenated monoterpenes, oxygenated diterpenes constitute 36.1%, 31.3% and 12.6%, respectively [112]. Fatty acid derivatives, aldehydes, alcohols, ketones, esters and others also exist in essential oils.

## 3. Sample Preparation

Sample preparation is the first challenge in analyzing *Gentiana* species, and is required for the availability of pure samples for subsequent study [113]. A suitable and efficient sample preparation method is especially challenging due to chemical diversity. Further compounding the challenge of sample preparation, pretreatment methods can adversely impact a plant’s chemical composition. There is an obvious case to illustrate: The first alkaloids to be discovered were mistakenly considered the main active ingredient of Gentianae Macrophyllae Radix. ChP (1977) recorded alkaloids as the standard of chemical and physical testing. However, in 1983, Guo and Lu proved that Gentianae Macrophyllae Radix did not, in fact, contain alkaloids. Alkaloids were formed due to the addition of ammonium hydroxide when the sample was treated [114]. Subsequently, many studies have shown that alkaloids are an artificial product in *Gentiana* species [115]. The methods of extraction in *Gentiana* species are summarized in Table 2, which describes the characteristics of different extraction techniques.

### 3.1. Extraction Methods

#### 3.1.1. Volatile Oils

Heat-reflux extraction (HRE) [83], steam distillation or hydrodistillation (HD) [29,108] and simultaneous distillation-extraction (SDE) [9,116] are conventional methods in volatile compound extraction. SDE uses a combination distillation-extraction apparatus for small quantities of plant material in a less time-intensive extraction and utilizes volatile extraction and distillation simultaneously. This method is considered superior to HD [118,119,120,121]. Monoterpenes are so unstable that chemical changes can be caused under steam distillation; some volatile compounds are lost when solvent is removed by distillation [119]. An alternative to traditional headspace sampling is solid-phase micro extraction (SPME), which is solvent-free, rapid, automatic, user-friendly, inexpensive and sensitive to odor analysis [122,123]. HS-SPME has a particularly good analytical efficiency (more than 100 compounds were detected) in only 20 min by consuming 2 g of herbs [112]. The operation process of SPME is as follows: The SPME device includes a holder and SPME fibers. The sample is placed in a headspace vial. The SPME device is exposed to the headspace for volatile component extraction. The extraction process requires a constant temperature and time. Therefore, the SPME fibers, time and temperature can be optimized for good analytical efficiency. Mustafa et al. studied this optimization process. Finally, compared to traditional methods, automated and efficient HS-SPME is a very promising and widely available extraction method for volatile oils [111].

Volatile oils are mostly extracted using distilled water. Other organic reagents like methanol are utilized. The volatile oil can also be purified using a preceded by liquid–liquid extraction as follows: methanol–chloroform, ethyl acetate, *n*-butanol [29]; water–diethyl ether [74]; water–*n*-hexane, *n*-hexane/chloroform (1/1), chloroform [109]. The oil yield can exceed 6.95% (% from the fresh weight) using HD for leaves of *G. asclepiadea* [108]. 

#### 3.1.2. Non-Volatile Compounds

The extraction of non-volatile compounds is performed by different methods. For example, soak extraction requires maintaining the sample at room temperature for 24 h [124]. HRE needs 1.2 h per extraction, and multiple extractions must be performed for optimal results [62,117]. Shaking extraction (SE) requires a mechanical shaker at 150 rpm for 2 h [116]. Soxhlet extraction requires a long extraction time, usually exceeding 2 h [27,125]. The main disadvantages are the long time required to obtain ideal results and the large volumes of solvents needed to perform the extractions. In order to improve efficiency of extraction, ultrasound extraction (UE) [52,63,126], ultrasound-assisted extraction (UAE), smashing tissue extraction (STE) [94], microwave-assisted extraction (MAE) [53], solid phase extraction (SPE) [30], and accelerated solvent extraction (ASE) [54] have been applied in extraction. SPE can be considered as a simplified version of column chromatography used for the extraction of iridoids and flavonoids in the water extracts of *G. algida*, *G*. *decumbens*, *G. macrophylla*, *G. triflora*. Relatively simple sample processing methods were used [30]. ASE is a new rapid technology that uses less solvent. The methanol extraction of *G.* lutea was completed in 10 min [54].

Moreover, organic solvents of different polarities are applied in the extraction process including methanol, ethanol and different proportions of methanol/ethanol-water mixture. According to the large amount of literature data, methanol is the most extensive solvent for obtaining the most ingredients, including iridoids and secoiridoids, xanthones, triterpenoids, flavones. From the literature, high proportions of organic solvents (>65%) are generally able to satisfy the analysis of most types of compounds. For example, the extraction rates of compounds **4**, 65% ethanol, 75% ethanol, 55% ethanol, 45% ethanol were 14.53%, 14.42%, 13.26% and 12.50%, respectively. Among them, the 65% ethanol concentration resulted in the highest extraction rates [124]. In another study, water, methanol, 20%, 40%, 60%, 80% ethanol aqueous were tested for extraction of gentisides A, B, J, K. The ethanol aqueous solutions of proportions below 80% could not dissolve these compounds, whereas 95% ethanol was a good extraction proportion [91]. Wei et al. also tested methanol, aqueous methanol and ethanol as solvents for extracting the iridoids and secoiridoids. Results show that methanol allowed complete extraction of ten iridoid and secoiridoid constituents [47]. Oleanolic acid, which is a triterpenoid, has been extracted for analysis by methanol [87]. A 70% methanol aqueous solution was used for simultaneous extraction of compounds **1**, **4**, **8**, **9**, **17** and ferulic acid (iridoids and secoiridoids, xanthones, triterpenoids) from *G. rhodantha*, *G. farreri*, *G. scabra*, *G. rigescens* [49]. However, low proportion of organic solvents (<60%) is suitable for the extraction of 4-pyrones (compound **17** as representative) [15]. Moreover, enzyme treatment increased the total yield of maceration by 3.5%, but the concentration of bitter compounds did not increase [116].

In the extraction process, temperature is another often mentioned parameter. HRE, Soxhlet use a heating process in the extraction. Under higher temperatures, components may be destroyed. It can be assumed that this is caused by an unstable chemical structure. At 180 °C, the extraction rate of MAE was lower than at room temperature [47]. According to the literature, most extraction is carried out at room temperature and 40–60 °C [21,44,46,48,52,55,63,65,78,80,82,83,90,101,103,127]. A temperature of 100 °C is generally used for water extraction of polysaccharides [95]. From these reports, it is not difficult to determine that moderate temperature is suitable for most ingredients. Response surface methodololgy (RSM) and design-based Central composite design (CCD) are used to optimize the extraction of gentian total glycosides and polysaccharides [17,53,94,128].

Different analyses of targets, including trace elements, free amino acids, and polysaccharides, require different preparation methods. Microwave assisted digestion (MAD) employs different acid mixtures and microwave heating systems in the determination of elements [98,99,129]. In amino acid analysis, due to no significant fluorophores and presence of interference from impurities in a complex sample mixture, a fluorescence derivatization procedure was introduced into the analysis for providing strong chromophores or fluorophores and facile detection in an HPLC-FLD system [100,101,130]. Usually, as a general principle of polysaccharide extraction, hot water is used as a solvent in order to avoid the effects of some impurities. The sample was first degreased with petroleum ether or ethanol in a reflux apparatus or soxhlet apparatus. Defatted samples were treated with ethanol or 80% ethanol; subsequently the ethanol was removed and the insoluble residue was collected for HRE, UAE, MAE, STE. The polysaccharide precipitate was deproteinated by a sevage method, a combination of proteinases treatments and sevage method or polyamide adsorption method. Further purification and separation of crude polysaccharides was usually done by centrifugation and column chromatography [92,93,94,95,128]. According to the study applied by Cheng et al. three common extraction techniques (HRE, UAE, MAE) and a novel STE technique were recommended to extract polysaccharides from *G. scabra*. The yield obtained from STE (15.03 ± 0.14%) was the highest among the four methods, followed by MAE, HRE and MAE. In addition, the author compared average molecular weight and antioxidant activity of polysaccharides. These results implied the antioxidant capability of polysaccharides was affected significantly by the extraction method. STE gave the highest extraction yield with the highest antioxidant ability in the shortest extraction time [94]. Table 3 shows the extraction rate of different extraction methods for volatile and non-volatile compounds.

### 3.2. Separation Technologies

In recent years, main separation technologies have been optimized for phytochemical considerations: column chromatography, semi-preparative (semi-prep) and preparative (prep) HPLC and high-speed counter-current chromatography (HSCCC). Preparative TLC and droplet counter-current chromatography (DCCC) methods have rarely been reported.

Despite recent advances, classical column chromatography is still widely used. Samples of crude extracts were further purified on silica gel [54,131], macroporous resin (ODS) and polyamide adsorption column [66] with various solvents. Sometimes, the further purification steps are present in preparative reversed-phase HPLC or another separation technology like HSCCC. 

Crude polysaccharide from *G. rigescens* was further fractionated on ODS and eluted with deionized water, followed by 30%, 60% and 90% ethanol to obtain a fraction 1 which was found to have good antiviral activity against human respiratory syncytial virus [95]. The *n*-BuOH extract of *G. piasezkii* was subjected to column chromatography on resin and silica gel to yield five flavonoids [84]. A total of 7 iridoids and secoiridoid glycosides were isolated from *G. triflora* by a combination of ODS followed by semi-preparative HPLC [66]. Preparative TLC is used to separate compound **4**. The spots were scraped off from preparative TLC and then subjected to HPLC analysis [115].

Compared to classical column chromatography, the separation time of the DCCC method is relatively shorter and less solvent is consumed. The DCCC method had been used to isolate xanthone-*O*-glucosides (1,3,5-trihydroxy-xanthone-8-*O*-β-d-glucoside (the yield was 53.3%) and 1,5-dihydroxy-3-methoxy-xantbone-8-*O*-β-d-glucoside (the yield was 43.3%) from the crude extracts of *G. strictifrora*. The flow rate and time of separations were 10–15 mL·h^−^^1^, 23–35 h, respectively. Moreover, the more polar layer could result in a shorter separation time but resolution would be poor. This confirms that DCCC is suitable for separating glycosides and other polar compounds [132,133]. However, compared to HSCCC, DCCC requires a long separation period and high solvent consumption. For instance, compounds **4**, **9**, **8**, **1** and **27**, **5** were separated simultaneously from *G. crassicaulis* through HSCCC with *n*-butanol/ethyl acetate/methanol/1% acetic acid water (7.5:0.5:0.5:3.5). The flow rate and time of separations were 102 mL·h^−^^1^, 7.5 h, respectively. The highest isolation rate was 8.14% for compound 4 [53]. Another study reported that a total of 7 components were separated from *G. macrophylla* through combining HSCCC with preparative HPLC. Among them, 3 compounds (deglucoserrulatoside, compounds **1** and **25**) were isolated by HSCCC using a ethyl acetate/*n*-butanol/H_2_O (2:3:5:0.6) solvent system. Subsequently, through purification of semi-preparative HPLC from Fra2, Fra3, and Fra4, which were produced from HPCCC, Compounds **4**, **9**, **8** and **42** were obtained. Besides, Compounds **4**, **8**, **1** and **42** of the extracts of *G. macrophylla* were also successfully isolated by HSCCC using an *n*-butanol/0.1%aqueous trifluoroacetic acid (1:1) system [60,134]. A one-step HSCCC method was established to separate 8-hydroxy-10-hydrosweroside, compound **8** and **15** from the crude extract of *G. scabra*. This method utilized a two-phase solvent system consisting of *n*-hexane/*n*-butanol/methanol/0.4% acetic acid in water (1.4:8:3:15.5, *v*/*v*) [135]. Obviously, HSCCC is very versatile and facilitates separation. Its advantages have been reported in the literature [136,137]. Further, a solid stationary phase is not required, and ameliorates can be irreversibly adsorbed, with short analysis time, and no need for expensive columns [113,138]. Traditional column chromatography is used for the fractionation of crude plant extracts (in multigram quantities) or for final purification steps.

## 4. Analytical Methods

**Figure 3:**
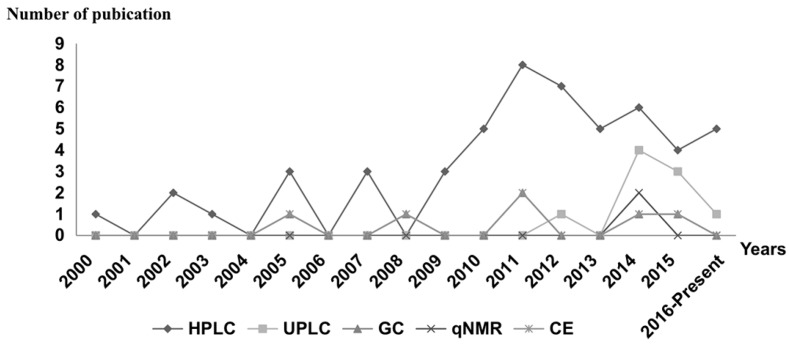
The development trend of the main analysis methods.

Research on the material properties is necessary for pharmacological activity and quality control, as well as the taxonomy of related plants. Various analytical methods for *Gentiana* species are listed in Table 4, including sample information and preparation, as well as detection conditions. In addition, Figure 3 shows the development trend in HPLC, UPLC, GC, qNMR, CE from 2000 to 2016–present.

### 4.1. Spectroscopy

Spectroscopic methods, including infrared (IR), mid-infrared (MIR) and near-infrared (NIR), atomic absorption spectroscopy (AAS), and inductively coupled plasma-atomic emission spectroscopy (ICP-OES), have been used for the analysis of *Gentiana* species. Among them, AAS and ICP-OES are commonly used for elemental analysis.

#### 4.1.1. Infrared (IR)

In the genus *Gentiana*, the application of IR is mainly divided into two classes. On the one hand are qualitative and quantitative assays; on the other hand is a classification of different samples. Quantitative analysis of compounds **1** and **3** was presented in tissue culture samples of different stages of *G. scabre*. Complex correction models and different spectral pretreatments were established in order to accurately quantify. The reference values were measured for two compounds through HPLC. This method showed a fast and accurate result [67,89]. Owing to chemometrics that expand the use range of IR, this combination was applied to the clustering of different samples. However, hierarchical cluster analysis or principal component analysis (HCA/PCA) and FT-IR spectra failed to identify some chemical information and in monitoring, especially for the content of individual metabolites from complex data matrix, some problems still exist. For instance, raw and processed products of *G. rigescens* (wine-, vinegar- and salt water-processed) showed a dissatisfactory classification performance. Tissue culture samples of different stages showed a significant classification, whereas the detailed variation failed to be monitored. This problem was solved through LC [107,140]. FT-IR spectroscopy combined with PCA and partial least squares discriminant analysis (PLS-DA) or IR fingerprinting was also used to distinguish different origins of *G. rigescens* and *G. macrophyllae*, respectively [139,152]. It is obvious that these methods are very complex and require professional software as well as personnel to complete spectral pretreatments and modelin*G.* Although, specificity, sensitivity, resolution, trace and multi-component detection are limited in detection, the rapid, nondestructive characteristics and simple sample preparation are beneficial to anaysis of *Gentiana* species.

#### 4.1.2. Atomic Absorption Spectroscopy (AAS) and Inductively Coupled Plasma-Atomic Emission Spectroscopy (ICP-AES/OES)

Mineral elements not only play a key role in the formation of active components and in biological function, but also in nutrient content. The concentration of elements is an indicator of whether they are beneficial or harmful to the body. At the same time, heavy metals present an index of quality evaluation. Through the AAS method, a total of 9 elements (K, Ca, Na, Mg, Fe, Cu, Zn, Se and Cr) were determined in *G. rigescens* [97]. Mn, Zn, Cu, Co, Cr, Pb, Ni and Cd were determined in *G. lutea* [97]. Different elements require different detection wavelengths. Reference substances and standard curves are needed in quantitative analyses. Compared to AAS, ICP-AES and ICP-MS provide a wider range of detection and simultaneous detection [153]. For example, 18 elements (Al, As, B, Ba, Ca, Cd, Co, Cr, Cu, Fe, K, Mg, Mn, Na, Ni, Pb, Sr and Zn) were simultaneously detected in *G. lutea* together with soil samples [98]. Ca, Na, Zn, Cu, Mn, Fe, Mg, K, P, and B were compared between flowers and roots of *G. macrophylla* [99]. AAS and ICP need complex sample pretreatment and standard substances. However, for the latter, simultaneous detection is the greatest advantage.

### 4.2. Thin-Layer Chromatography Analysis (TLC) and High Performance Thin-Layer Chromatography (HPTLC)

TLC is a simple, convenient, fast, and low cost alternative to routine chemical analysis. As a rapid qualitative analysis tool, TLC is considered a ChP and JP standard. Moreover, TLC also is used in a variety of analysis processes. For instance, detecting ballast substances washed using column chromatography [154], simple chemical analysis in a pharmacological study [4] and as a supplementary technology for qualitative analysis [51,83]. Unfortunately, the limitation of separation capabilities, sensitivity and reproducibility make it difficult to quantify and analyze multi-components. HP-TLC as a refined version of TLC provides a solution. In *G. lutea*, compound **1** was quantitatively determined at 280 nm absorption wavelength through TLC-densitometry. This process used CH_2_Cl_2_:MeOH:H_2_O (39:10:1) and a silica gel 60F254 plate to improve the resolution and reproducibility compared with silica gel 60 plate [68]. Hayashi and co-workers also developed a similar method which used CHCl_3_:MeOH:H_2_O (30:10:1) and separation on a Wakogel b-5fm plate for the determination of compound **1** in *G. triflora var*. *japonica*, *G. scabra var*. *buergeri*, Gentian radix, *G. scabra* radix and *G. scabra* Bunge [69,70,71]. Currently, TLC is a less common technique for quantitative analysis. Technological innovation of TLC, such as two-dimensional TLC or HPTLC-MS, has not been applied to chemical analysis of *Gentiana*. However, it should be noted that due to the advantages of HPTLC-MS, such as high selectivity and efficiency, low detection limits, fast separation times, it might have a promising future for quantitative analysis.

### 4.3. Gas Chromatography Analysis (GC)

Gas chromatography (GC) is commonly employed to determine volatile compounds. The advantage of GC is high resolution for detection of volatile compounds. Also, GC can be utilized for analysis of nonvolatiles such as endogenous gibberellins with a derivatization process. The flame ionization detection (FID) and capillary columns (commonly fused-silica) are used in most GC experiments. Arberas et al. analyzed the complex aroma of the fresh roots and rhizomes of *G. lutea*, as well as those of two subspecies. A total of 83 components were identified through GC combined with FID, flame photometric detector (FPD), infrared detector and a mass selective detector. In addition, GC-Olfactometric (GO-O) and GC/FID collocation were used to describe the fundamental characteristics of the key fragrant compounds [9]. With the appearance of mass spectrometry (MS), GC-MS is widely used for the detection of volatile components. The GC-MS experiments are performed using electron ionization (EI). Jaemin et al. characterized the floral scent of 13 cultivars of gentians through HS-SPME coupled to GC-ESI/MS [110]. Different species, various parts, wild vs. cultivated, and commercial products were all analyzed by GC-MS [29,88,108,109,111,112]. Except for analysis of volatile components, GC-MS was used to identify endogenous gibberellins (GAs) in vegetative growth stages of *G. triflora*. GAs were determined with derivative reagents and subjected to analysis [144]. For determination of volatile oils and neutral ingredients in *Gentiana* species, GC is a powerful separation technique. On-line detection allows accurate qualitative and quantitative determination. The introduction of HP-SPME and MS improves the efficiency. Overall, GC has more advanced and complete mass spectral databases (e.g., the National Institutes of Standards and Technology) than LC-MS. This makes data processing easy and metabolomic analysis fast. With the development of two-dimensional chromatography, some advantages compared with one-dimensional gas chromatography have been reported, such as increased selectivity and peak capacity; enhanced sensitivity; and increased identification power (e.g., fatty acid methyl esters). In addition, GC × GC can also use detection systems, such as a mass spectrometer [155]. Thus, two-dimensional (2D) gas chromatography (GC × GC) could be used as a powerful analysis technique for scientific research of *Gentiana* species, although, this analytical technique is not fully demonstrated in *Gentiana* species.

### 4.4. High-Performance Liquid Chromatography Analysis (HPLC)

HPLC is the most commonly used method for qualitative and quantitative analysis as well as fingerprint chromatography of *Gentiana* species. Quantitative analyses of ten iridoids and secoiridoids in the roots of *G. straminea* were performed using HPLC-UV at 254 nm for 60 min [47]. Generally, this analysis process is carried out on reversed-phase C18 columns (150 or 250 mm × 4.6 mm × 5 μm) with an isocratic or gradient elution mode. Water or acidic water (phosphoric acid, formic acid, acetic acid, trifluoroacetic acid and phosphate buffer) and acetonitrile, *n*-propanol or methanol mixtures are used as two-phase solvent systems. The solvent range (1% to 100%) is adjusted to obtain an appropriate gradient elution program. Table 4 shows the detailed chromatographic conditions. According to the results of optimization of chromatographic conditions, acid water is often chosen because it can enhance resolution, eliminate peak tailing and enhance ionization in mass spectrometry [45,49,91]. Compared to other analytical techniques (like, IR, qHNMR, CE), HPLC shows the following advantages: high sensitivity and reproducibility, good resolution and linearity, ease of automation and multi-component analysis [156]. The application mostly regards quality control, material basis, metabolite accumulation, and resource development. For example, HPLC is used alone or combined with other technology like DNA bar coding and ISSR-PCR for quality control of different *Gentiana* species [56,145]. Fingerprint analysis is also available in quality identification [46,52] and investigation of adulterants [23] or substitutes [45]. In pharmacological research, the constituents in the extracts were analyzed by HPLC-UV or HPLC-MS [57,72,80,81,103,105,157].

The advantages of HPLC are obvious but the analysis time is too lon*G.* From the literature, the analysis time is more than 30 min. In comprehensive monitoring or analyzing a large number of compounds, the time reaches 120 min. Sheu et al. investigated the flavonoids and phenolic acids in *G. macrophylla* root extracts. In order to completely separate 33 components by HPLC-DAD, the analysis time was 120 min [146]. Recently, Olennikov et al. significantly improved the analysis time by using microcolumn-RP-HPLC-UV: 13 compounds of iridoids and phenolic were separated in 4 min at 230 nm and 334 nm wavelength, respectively. This method uses a small particle size column (1 mm × 50 mm × 1 μm) and shows a very small amount of injection and low flow rate (1 μL, 600 μL·min^−1^). The run time and solvent consumption are thus reduced, which results in more environmentally friendly and economic analysis [30]. Another technique uses an ultra performance liquid chromatography (UPLC) and ultra-fast liquid chromatography (UFLC) system with a 150 mm × 2.0 mm × 2.2 μm or 75 mm × 2.0 mm × 1.6 μm column. The analysis time is mostly within 30 min without a reduction in resolution [17,49,126,148,149]. For instance, fingerprint analysis of the seed, root, stem, leaf and flower of *G. rigescens* as well as quantification of metabolites exhibited satisfactory performance in 19 min by UFLC-UV-MS/MS [107,148].

#### 4.4.1. HPLC Coupled with Conventional Detection

Ultraviolet (UV/Vis) detection and diode array detection (DAD or PDA) are the most widely used detectors. A large number of studies have reported qualitative and quantitative analysis of chemical compounds by using these two detectors. Both are readily available in the laboratory at low cost. UV and DAD detectors provide a discrete range of wavelengths (190–450 nm) for qualitative and quantitative analysis according to the retention time and peak area. They are capable of monitoring several wavelengths and recording on-line by applying a multiple wavelength scanning program and comparison of UV structure information with the standard. If present in adequate quantity, all UV-absorbing compounds are detected. Schaufelberger and Hostettmann analyzed secoiridoid glycosides and flavonoids in *G. sino-ornata*, *G. lawrencei* and their hybrid by HPLC-DAD. DAD detection was respectively set at 240 nm and 254 nm. Although the UV spectra of flavonoids were similar, shift reagents were added to the eluent to improve the characterization of polyphenolics [73]. Other detectors, including evaporative light scattering detection (ELSD), refractive index detectors (RID) and fluorescence detectors (FLD) are also used in the analysis of free fatty acids and polysaccharides, but reported less. In a study on the two new methods, iridoid glycosides were isolated from the roots of *G. dahurica*, in order to elucidate the chemical characteristics of the hydrolysis product. HPLC-ELSD was employed for the detection of d-glucose [77]. The purity of polysaccharide was tested by HPLC-RID [95]. Free fatty acids were successfully detected in *G. straminea* and *G. dahurica* by the HPLC-FLD-MS system; nevertheless, the analysis process requires derivatization to improve stability, optimize recovery and separation, and enhance the detection [100,101].

#### 4.4.2. Liquid Chromatography-Mass Spectrometry (LC-MS)

The most modern analytical techniques are based on the MS and separated by LC (HPLC or UPLC). It has already occupied a very important position for chemical analysis. Contrary to the previously applied methods that reveal no structural information on analytes, the mass spectral of molecular ion and fragment information provide a reliable identification [158]. Working in multiple reaction monitoring (MRM) or selected reaction monitoring (SRM) mode can drastically decrease the background noise of a spectrum and will therefore have a positive influence on the signal-to-noise ratio which often leads to an increased sensitivity [159]. Obviously, these instruments are used not only for the identification but also for the quantitation of analytes. In a quantitative study of compounds of *G. lutea*, LOD and LOQ obtained by HPLC-ESI-MS were 10 times lower than those obtained by HPLC-DAD [11,12]. Another study conducted quantitative analysis of *G. rhodantha*, *G. farreri*, *G. scabra* and *G. rigescens*. The presence of ferulic acid in *G. farreri* was validated by comparing the retention time and UV spectrum data of the standard, while the above conclusion was denied when reconfirmed by MS data [49]. Remarkably, the MS proved to be more sensitive and accurate than the UV or DAD detection. In some literature, LC-MS and LC-UV (or DAD) are used separately. Compared with the analytical methodologies, LC-MS was regarded as qualitative authentication. LC-UV/DAD was used for quantitative and fingerprint analyses [48,52,54]. In most of the literature, MS can be easily used in tandem with a conventional detector to form an LC-DAD-MS system for qualitative and quantitative analysis. As proof of principle, in anti-oxidative experiments, active constituents (compounds **4**, **9**, **8**, **1**) of *G. cruciata* were quantified by UPLC-DAD-HESI-TSQ-MS under SRM mode [102]. HPLC-DAD-ESI-MS was used for quantitative determination of phenolic compounds and iridoids in anti-inflammatory experiments of *G. macrophylla*, *G. dahurica* and *G. straminea* [40,78]. Simultaneous qualitative and quantitative determination of ten iridoids and secoiridoids was executed in the HPLC-UV-ESI-MS system for quality control of *G. straminea* [47].

Ion trap mass spectrometry (ITM), triple quadrupole mass spectrometer (QQQ/TSQ), time of flight (TOF), quadrupole-orbitrap (Q-Exactive) and quadrupole-TOF (Q-TOF) are currently the main uses of mass spectrometers. Electrospray ionization (ESI) and atmospheric pressure chemical ionization (APCI) are the ionization methods of choice in chemical analysis. Among them, APCI is used for qualitative identification of the amino acids in *G. straminea* and *G. dahurica*. In the application of these mass spectrometers, ESI-ITM, Q-Exactive, QQQ and TOF were used for non-target or target screening, and no quantitative data were acquired. Fragmentation pathways for identification of unknown compounds were elucidated [23,105,147,148,160]. Additionally, these experiment procedures could be aided by QTOF-MS [151]. QQQ with MRM mode was used for the quantitation of targeted analytes. The group of Pan et al. used this method for the qualitative and quantitative analysis of different *Gentiana* species [49,91,107,140,148]. According to the literature on *Gentiana*, there is no comparison of the advantages and limitations of each mass spectrometer. In contrast with LC-UV/DAD, the results obtained with MS are not congruent but rather instrument dependent. Therefore, choosing the right mass spectrometer for a particular study is even more important. Further, these mass spectrometry instruments can be used together or alternately. Finally, LC-MS with a particular advantage can be widely applied in quality control and chemical analysis. But the generation of useful and reliable data depends on the experience of the operator. In addition, a lot of time and work are needed to analyze and construct standard mass spectrum libraries of a large number of analytes.

### 4.5. Quantitative 1H Nuclear Magnetic Resonance (qHNMR)

In the genus *Gentiana*, a relatively underexplored analytical method-qHNMR is applied for quantitative and purity analysis. This method requires the selection of an internal standard and targeted signals. By comparing the signal integration to internal standards, the quantitative result was easily obtained (the ratio of the signal integration was proportional to the concentration). Compound **4** was quantified in the crude methanol-*d*_4_ extract of *G. scabrae* and Gentianae radix. Hexamethyldisilane was selected as an internal standard. The result was verified through HPLC. Under the condition that the signal was independent, the result was slightly higher than that of HPLC [141]. 1,4-dinitrobenzene was used as the internal standard for assessing the purity of iridoids and secoiridoids. The result was also verified via HPLC [142]. This experiment concluded that the qHNMR method is reliable but the selection of quantification signal and internal standard is relatively complex. Simultaneously, to obtain accurate quantitative results, there should be no overlapping signals. ^1^H-NMR spectra, 2D COSY and HMQC spectra, therefore, were measured in order to confirm the presence or absence. In short, the analysis process of qHNMR is fast (run time: ca. 6 min), and it does not consume solvents and reference compounds (or calibration curves). Simultaneous determination of multi-components can also be achieved. Compared to LC, sample processing is relatively simple.

### 4.6. Capillary Electrophoresis (CE)

The CE method requires low solvent and sample consumption (microgram per litre) and is fast. For example, Quantitative analysis of compounds **10**, **19**, **18** in *G. lutea*, CE (11 min) enables significantly improved separation time compared to HPLC-UV (30 min). However, a comparison of the LOQ showed poor sensitivity [90]. Considering the advantages, it is used for the analysis of compounds.

Micellar electrokinetic capillary chromatography (MEKC) and UV [74,75] are main separation modes and detectors. Capillary zone electrophoresis (CZE) [86] and DAD [90] also are used. Compounds **1** and **3** in *G. rigescens* were detected through MEKC [74]. Five phenolic compounds (7-*O*-feruloylorientin, 6′-*O*-vanilloylarbutin lutonarin, isoorient and luteolin) in *G. piasezkii* were determined through CZE [86]. These experiments detected a small number of analytes. Combined with MS, the structural information and selectivity provided an effective solution. Takahashi et al. investigated the metabolite profiles of the effects of K and P on *G. triflora* by CE-MS [143]. In general, the application of CE is limited in some respects, such as the difficult choice of background electrolytes and inferior repeatability compared with LC. This method mainly is used to analyse charged molecules.

## 5. Chemometric Analysis

Advanced analysis instruments have gradually resulted in datasets becoming larger and more intricate, often resulting in poor data processing and interpretation. However, the advent of chemometrics has made it possible to deal with such massive data. The term “chemometrics” was coined by Svante Wold in a 1971 grant application, which defined the field as the science of extracting information from chemical systems by data-driven means [161]. Today, chemometric tools have become crucial for extracting valuable information from raw data. Several reviews pertaining to the application and theoretical background of chemometrics have been published [162,163,164]. In this review, the application of chemometric combined with the chromatographic fingerprint of *Gentiana* species was discussed. The application of chemometric analysis of the developed chromatograms is summarized in Table 5.

Raw data from IR are very complex. In order to eliminate the spectral variation that is not caused by chemical information contained in the samples, chemometric preprocessing methods are essential in chromatographic fingerprint studies. Different pretreatments including multiplicative scatter correction (MSC), first or second derivative (FD or SD), Savitzky-Golay (SG) filter, and Norris derivative (ND) filter have been applied. MSC as an alignment technique was included to eliminate the NIR spectral variation caused by non-uniform particle size in a quantitative study of *G. scabra*. After MSC treatment, the spectra were subjected to smoothing; smoothing with FD; smoothing with SD in order to analyze the best pretreatment parameters of the analytes. After such a correction, similarity analysis and chemical pattern recognition can be completed for different studies. For instance, MPLSR, SMLR and ICA are used separately to establish spectral calibration models for quantitative analysis (predict the concentration of compounds **1** and **3**) [67,89]. HCA, PCA or PLS were employed separately to classify *G. rigescens* according to its geographical origin or growing stage [107,152], and classify different *Gentiana* species [45]. In a study of metabolite analysis, the above method can also reveal significant differences between various samples. In the absence of K and P cultivation conditions, CE-MS metabolite profiles of 47 metabolites of *G. triflora cv*. *Albireo* could be divided into a series of clusters. HCA and PCA were used to explain the principal components and the salient characteristics of each metabolite group [143]. With the help of SA, HCA, heat map and PLS-DA, the chemical diversity of *G. rigescens* and *G. rhodantha* in different plant parts and geographical origins was investigated based on metabolite fingerprinting of UFLC-UV-MS/MS. Potential markers were screened according to VIP values, a PLS-DA-based result, for discrimination of different geographical origins samples [17,148]. The same chemometric analysis was used in chemotaxonomic studies on the genus *Gentiana* or Swertia. Based on FT-IR and content of secondary metabolites from UPLC-QQQ-MS/MS data, SA, HCA, PCA and PLS-DA were used to rapidly find the chemotaxonomic marker [50]. 

Obviously, this combination strategy has made a significant impact on quality evaluation of *Gentiana*. More in-depth research can be done through this method, including evaluation and authentication of the quality, evaluation of the therapeutic effects and possible action mechanism. However, with the tremendous advances in analytical techniques and complex and difficult to understand theoretical background, dealing with massive complex and high-dimensional data is still a massive challenge

## 6. Conclusions

In this review, we summarized the existing studies on the chemical analysis of *Gentiana* species. According to the literature, 42 compounds were detected and up to 11 compounds were simultaneously quantitatively detected. Most of the studies were devoted to the analysis of iridoids, as represented by 54% of the publications. These compounds are considered as representative ingredients for quality assessment and identification. Among these compounds, different classes were described: 16 iridoids, 10 flavonoids, 8 xanthones, 1 triterpenoids and 7 others. Moreover, volatile oils and polysaccharides have been studied in terms of their composition or bioactivity. The study found that the secondary metabolites were affected by hereditary and the geographical environment. For instance, different species and hybrids show different chemical profiles, and cultivation conditions or environments such as altitude and soil pH have effects on secondary metabolites. Recently, advanced sample preparation techniques, for example, enrichment efficient SPME, and simple and fast SPE, ASE, STE, were used for qualitative and quantitative analysis. As for separation techniques, in addition to HPLC, which was widely used due to versatility, generalized availability and simplicity, LC-MS has recently been successfully used in qualitative and quantitative analysis of *Gentiana*. The application of UPLC and microcolumn-LC make up for the shortcomings of HPLC analysis in terms of length of time high solvent requirements. Furthermore, the application of LC-MS coupled with chemometrics has allowed better management of the larger and comparatively more intricate datasets produced by advanced analysis instruments, providing a new, low cost, less time-consuming, effective, comprehensive strategy for data analysis. 

From the current review, the present research on the analysis of *Gentiana* is not sufficient. Future research should pay attention to several aspects:

(1) According to the respective, analytical chemistry of the genus *Gentiana* is mainly concentrated on the following plants: *G. macrophylla*, G. *straminea*, *G. crassicaulis*, *G. dahurica*, *G*. *scabra*, *G. triflora*, *G. rigescens*, *G. manshurica*, *G. lutea*, *G. rhodantha*, *G. cruciata*, *G. farreri*, *G*. *officinalis*, *G*. *triflora*, *G. asciepiadea*, *G. olivieri*, *G. kurroo*, and *G. punctat*, and polysaccharides analysis are limited to *G. scabra* and *G. rigesens*. Compared with the whole *Gentiana*, the bulk remains unexplored.
(2) The analysis of triterpenoids and pesticide residue did not cause concern. Sample pretreatments still make use of outdated HRE and UA. In addition, polysaccharide research results inferred that different extraction methods can affect the activity of the extract, whether or not such an inference is limited to polysaccharides. Advanced extraction techniques must be carried out in a comprehensive and comparative study of extraction time, extraction yield, and bioactivity. The accumulation of flavonoids, xanthones and iridoids has significant differences during flowering and dormancy. Therefore, the proper time of plant collection should be taken into account.
(3) At present, due to the improvement of sample pretreatment methods and analytical instrumentations, establishment of innovative procedures for detecting compounds in materials becomes more effective. It is evident although the mechanism of the traditional clinical efficacy of *Gentiana* species is unclear. The understanding of phytochemical profile, bioactivity screening, biosynthetic pathway, and structure-activity relationship could be solved by innovative analytical strategies in further research.
(4) In the pharmacopoeia standard, the limitations of the genus *Gentiana* species can be observed. A single ingredient as the standard for quality control is insufficient, for instance, compounds **1**, **4**, and mangiferin are standards widely distributed in the genus *Gentiana*, so the detection standards are not representative. With the support of modern analytical instruments, the establishment of harmonious and effective medicinal herb standards needs integration of multi-disciplinary technologies like analytical chemistry, biology, chemometrics, etc. The improvement of quality criteria requires alignment with pharmacological activity. Fingerprinting and chemometric analysis can evaluate quality from a single or several ingredients to the whole. Overall, the immense therapeutic potential and application value of *Gentiana* can be evolutionary amplified on the basis of quality control in the future.

## Figures and Tables

**Table 1:** The molecular formula, weight and origin of all analytes.

No.	Compound Name	Molecular Formula	Molecular Weight	Origin	Refs.
**Iridoids and Secoiridoids**					
**1**	loganic acid	C_16_H_24_O_10_	376.358	*G*. *macrophylla*, *G*. *crassicaulis*, *G*. *straminea*, *G*. *Lutea*, *G*. *dahurica*, *G*. *waltonii*, *G*. *ihassica*, *G*. *scabra*, *G*. *officinalis*, *G*. *farreri*, *G*. *siphonantha*, *G*. *cruciata*, *G*. *manshurica*, *G*. *triflora*, *G*. *rigescens*, *G*. *rhodantha*, *G*. *purdomii*, *G*. *erectosepala*, *G*. *obconica*, *G*. *robusta*, *G*. *microdonta*, *G*. *decumbens*, *G*. *algida*, *G*. *lawrencei*, *G*. *crassicaulis*	[11,23,30,44,45,46,47,48,49,50,51,52,53,54,55,56,57,58,59]
**2**	loganic acid-6′-*O*-β-d-glucoside	C_22_H_34_O_15_	538.499	*G*. *decumbens*, *G*. *macrophylla*, *G*. *triflora*	[30]
**3**	deglucoserrulatoside	C_21_H_20_O_10_	412.479	G. *macrophylla*	[60]
**4**	gentiopicroside	C_16_H_20_O_9_	356.327	*G*. *scabra*, *G*. *lutea*, *G*. *dahuricae*, *G*. *triflora var*. *japonica*, *G*. *scabra var*. *buergeri*, *G*. *kitag*, *G*. *acrophylla*, *G*. *officinalis*, *G*. *siphonantha*, *G*. *rigescens*, *G*. *macrophylla*, *G*. *crassicaulis*, *G*. *straminea*, *G*. *dahurica*, *G*. *waltonii*, *G*. *ihassica*, *G*. *scabra*, *G*. *davidii var*. *formosana*, *G*. *cruciata*, *G*. *farreri*, *G*. *sino-ornata*, *G*. *“Macazdayi”*, *G*. *pneumonanthe*, *G*. *ascjepiadea*, *G*. *algida*, *G*. *lawrencei*, *G*. *manshurica*, *G*. *triflora*, *G*. *dinarica*, *G*. *veitchiorum*, *G*. *robusta*, *G*. *purdomii*, *G*. *erectosepala*, *G*. *obconica*, *G*. *microdonta*	[15,23,30,44,45,49,50,53,56,58,61,62,63,64,65,66,67,68,69,70,71,72,73,74,75,76]
**5**	6′-*O*-β-d-glucopyranosyl gentiopicroside	C_22_H_30_O_14_	518.468	*G*. *manshurica*, *G*. *scabra*, *G*. *triflora*, *G*. *rigescens*, *G*. *straminea*, *G*. *macrophylla*, *G*. *crassicaulis.*	[40,47,53,66]
**6**	6′-*O*-acetyl gentiopicroside	C_18_H_22_O_10_	398.364	*G*. *straminea*, *G*. *macrophylla*, *G*. *crassicaulis*, *G*. *dahurica*	[47,77]
**7**	3′-*O*-acetyl-gentiopicroside	C_18_H_22_O_10_	398.364	*G*. *dahurica*	[77]
**8**	swertiamarinin	C_16_H_22_O_10_	374.342	*G*. *straminea*, *G*. *macrophylla*, *G*. *algida*, *G*. *farreri*, *G*. *sino-ornata*, *G*. *“Macazdayi”*, *G*. *crassicaulis*, *G*. *lawrencei*, *G*. *rigescens*, *G*. *officinalis*, *G*. *lutea*, *G*. *cruciata*, *G*. *scabra*, *G*. *triflora*, *G*. *pneumonanthe*, *G*. *ascjepiadea*, *G*. *manshurica*, *G*. *rhodantha*, *G*. *kitag*, *G*. *acrophylla*, *G*. *dahurica*, *G*. *waltonii*, *G*. *officinalis*, *G*. *ihassica*, *G*. *siphonantha*, *G*. *scabra*, *G*. *davidii var*. *formosana*, *G*. *microdonta*, *G*. *robusta*, *G*. *erecto-sepala*, *G*. *dinarica*, *G*. *purdomii*, *G*. *erectosepala*, *G*. *obconica*	[15,23,30,40,44,45,48,49,50,53,63,65,66,73,75,76]
**9**	sweroside	C_16_H_22_O_9_	358.343	*G*. *straminea*, *G*. *macrophylla*, *G*. *algida*, *G*. *farreri*, *G*. *sino-ornata*, *G*. *“Macazdayi”*, *G*. *crassicaulis*, *G*. *lawrencei*, *G*. *rigescens*, *G*. *officinalis*, *G*. *lutea*, *G*. *cruciata*, *G*. *scabra*, *G*. *manshurica*, *G*. *triflora*, *G*. *pneumonanthe*, *G*. *ascjepiadea*, *G*. *rhodantha*, *G*. *dahurica*, *G*. *waltonii*, *G*. *officinalis*, *G*. *ihassica*, *G*. *siphonantha*, *G*. *microdonta*, *G*. *robusta G*. *asclepiadea*, *G*. *purdomii*, *G*. *erectosepala*, *G. obconica*	[15,23,30,40,44,45,48,49,50,53,66,73,76,78]
**10**	amarogentin	C_19_H_18_O_11_	422.342	*G*. *lutea*, *G*. *pannonica*, *G*. *punctata*, *G*. *purpurea*, *G*. *pneumonanthe*, *G*. *ascjepiadea*	[11,15,58,76,79]
**11**	2′-(*o*-*m*-dihydroxybenzyl)-sweroside 2′-*O*-(2,3-dihydroxyl-benzoyl)-sweroside	C_23_H_26_O_12_	494.449	*G*. *straminea*, *G*. *macrophylla*, *G*. *crassicaulis*, *G*. *rigescens*, *G*. *purdomii*, *G*. *microdonta*, *G*. *erecto-sepala*	[23,47,66]
**12**	amaropanin	C_29_H_30_O_12_	570.547	*G*. *pannonica*, *G*. *punctata*, *G*. *purpurea*	[79]
**13**	gentiotrifloroside	C_29_H_36_O_17_	656.194	*G*. *triflora*	[66]
**14**	rindoside	C_35_H_42_O_21_	798.221	*G*. *scabra*	[57]
**15**	trifloroside	C_35_H_42_O_20_	782.701	*G*. *scabra*	[57]
**16**	6′-*O*-(2-hydroxyl-3-*O*-β-d-glucopyranosyl-benzoyl)-sweroside	C_29_H_36_O_17_	656.590	*G*. *straminea*, *G*. *macrophylla*, *G*. *crassicaulis*	[47]
**Xanthones**					
**17**	mangiferin	C_19_H_18_O_11_	422.342	*G*. *lutea*, *G*. *asclepiadea*, *G*. *cruciata*, *G*. *triflora*, *G*. *rhodantha*, *G*. *farreri* ,	[15,30,49,50,80,81]
**18**	gentisine	C_14_H_10_O_5_	258.229	*G*. *pneumonanthe*, *G*. *ascjepiadea*, *G*. *lutea*	[48,76]
**19**	isogentisin	C_14_H_10_O_5_	258.229	*G*. *lutea*, *G*. *cruciata*	[48,58]
**20**	gentioside (isogentisin-3-*O*-β-d-primeveroside)	C_25_H_28_O_14_	552.485	*G*. *lutea*, *G*. *cruciata*, *G*. *dinarica*	[16,48,51,58,65]
**21**	gentioside isomer (1-hydroxy-3methoxy-7-*O*-primeverosyl xanthone)	C_25_H_28_O_14_	552.485	*G*. *lutea*, *G*. *cruciata*, *G*. *pneumonanthe*, *G*. *ascjepiadea*	[48,51,55,76]
**22**	7-hydroxy-3methoxy-1-*O*-primeverosyl xanthone	C_25_H_28_O_14_	552.485	*G*. *lutea*	[51]
**23**	norswertianin-1-*O*-primeveroside	C_24_H_26_O_14_	538.131	*G*. *dinarica*	[58,65]
**24**	1,6,8-triOH-3,5,7-triMeO-xanthone	C_15_H_12_O_7_	304.254	*G*. *lutea*	[54]
**Flavones**					
**25**	isovitexin	C_21_H_20_O_10_	432.381	*G*. *decumbens*, *G*. *macrophylla*, *G*. *triflora*, *G*. *algida*, *G*. *lutea*, *G*. *asclepiadea*, *G*. *crassicaulis G*. *officinalis*, *G*. *straminea*, *G*. *lawrencei*, *G*. *rigescens*	[15,30,50,78]
**26**	isoorientin (homoorientin)	C_21_H_20_O_11_	448.100	*G*. *decumbens*, *G*. *macrophylla*, *G*. *triflora G*. *algida*, *G*. *crassicaulis*, *G*. *officinalis*, *G*. *straminea*, *G*. *lawrencei*, *G*. *rigescens*, *G*. *olivieri*, *G*. *lutea*, *G*. *asclepiadea*, *G*. *cruciate*, *G*. *gelida*, *G*. *septemfida*, *G*. *piasezkii*, *G*. *farreri*, *G*. *sino-ornata*, *G*. *“Macazdayi”*	[15,30,40,50,73,78,82,83,84,85]
**27**	isoorientin-4′-*O*-glucoside	C_27_H_30_O_16_	610.521	*G*. *crassicaulis*, *G*. *farreri*, *G*. *“Macazdayi”*, *G*. *macrophylla*, *G*. *straminea*, *G*. *decumbens*, *G*. *triflora*, *G*. *algida*	[30,40,53,73]
**28**	isoscoparin	C_22_H_22_O_11_	462.407	*G*. *decumbens*, *G*. *macrophylla*, *G*. *triflora*, *G*. *farreri*, *G*. *sino-ornata and their hybrid*, *G*. *“Macazdayi”*	[30,73]
**29**	saponarin	C_27_H_30_O_15_	594.522	*G*. *piasezkii*, *G*. *decumbens*, *G*. *macrophylla*, *G*. *triflora*, *G*. *algida*	[30,84]
**30**	lutonarin	C_27_H_30_O_16_	610.521	*G*. *piasezkii*, *G*. *decumbens*, *G*. *algida*, *G*. *macrophylla*, *G*. *triflora*	[30,84,86]
**31**	isosaponarin	C_27_H_30_O_15_	594.522	*G*. *triflora*, *G*. *decumbens*, *G*. *linearis*	[30]
**32**	luteolin	C_15_H_10_O_6_	286.239	*G*. *piasezkii*	[84,86]
**33**	vitexin	C_21_H_20_O_10_	412.479	*G*. *macrophylla*, *G*. *straminea*	[40]
**34**	7-*O*-feruloylorientin	C_31_H_28_O_14_	624.551	*G*. *piasezkii*	[84]
**Triterpenoids**					
**35**	oleanolic acid	C_30_H_48_O_3_	456.711	*G*. *olivieri*	[87]
**Others**					
**36**	gentiside A	C_29_H_50_O_4_	462.715	*G*. *rigescens*	[88]
**37**	gentiside B	C_30_H_52_O_4_	476.742	*G*. *rigescens*	[88]
**38**	gentiside J	C_29_H_50_O_4_	462.715	*G*. *rigescens*	[88]
**39**	gentiside K	C_30_H_54_O_4_	490.769	*G*. *rigescens*	[88]
**40**	7(*R*)-*n*-butyl-morroniside	C_24_H_40_O_9_	472.575	*G*. *straminea*, *G*. *macrophylla*, *G*. *crassicaulis.*	[47]
**41**	7(*S*)-*n*-butyl-morroniside	C_24_H_40_O_9_	472.575	*G*. *straminea*, *G*. *macrophylla*, *G*. *crassicaulis*	[47]
**42**	macrophylloside D	C_25_H_34_O_14_	558.553	*G*. *macrophylla*	[60]

**Table 2:** Characteristics of different extraction techniques.

Technique	Core Technology	Extraction Time	Extraction Efficiency	Operation	Automation	Cost	Refs.
HRE	Heating	Long	High	Simple	Difficulty	Low	[47]
SE	Mechanical force	Long	Moderate	Simple	Difficulty	Low	[116]
Soxhlet	Soxhlet extractor	Long	High	Moderate	Possible	Low	[57]
UE/UAE	Ultrasound	Moderate	High	Simple	Difficulty	Moderate	[47]
MAE	Microwave	Short	Moderate	Simple	Difficulty	Moderate	[47]
STE	Mechanical shear power	Short	High	Simple	Possible	High	[94]
HD	Heating	Long	Low	Simple	Difficulty	Low	[29]
SDE	SDE apparatus	Long	High	Simple	Difficulty	Low	[116]
HS-SPME	SPME device	Short	High	Simple	Easy	High	[117]

**Table 3:** Yield of different extraction methods in *Gentiana* species.

Species	Parts	Extraction Method	Yield %	Time	Solvent	Refs.
**Essential Oil**						
*G. lutea*	Flowers	HD	2.70 *	4 h	Water	[108]
	Leaves		1.15 *			
*G. punctata*	Flowers		1.65 *			
	Leaves		4.2 *			
*G. asclepiadea*	Flowers		1.9 *			
	Leaves		6.95 *			
	Underground	Soxhlet	0.3 #	12 h	Methanol	[29]
	Aerial part		1.1 #			
*G. nubigenan*	Flowers	Soke + HRE	0.9 #	9 h	Water	[88]
*G. lutea*	Roots	HD	2.70 *	2 h	Water	[109]
**Non-Volatile Components**						
*G*. *lutea* ssp. *symphyandra*	Roots	Soak	49.4 *	/	Ethanol	[81]
*G. cruciata*	Aerial parts and Roots	Soak + stir	25 *	24 h (3 times)	Methanol	[102]
*G. olivieri*	Flower and Aerial parts	Soak + UE	/	150 min	methanol	[91]
*G. piasezkii*	Whole plant	HRE	10.75 *	60 min	80% methanol	[84]
		Soxhlet	49 *	4 h	methanol	[81]
		UE	5.71~28.05 *	30 min	Methanol	[63]
*G. lutea* ssp. *symphyandra*	Roots	UE	35.2 *	45 min	Methanol	[11]
*G. crassicaulis*	/	MAE	29.4 *	3.39 min	57.5% methanol	[53]
**Polysaccharide**						
*G. scabra*	/	STE	15.03 *	2.17 min	Water	[94]
		HRE	11.12 *	3 h		
		UAE	10.41 *	60 min		
		MAE	12.56 *	4 min		
		HRE	13.0 *	3 h		[128]

*: % of fresh weight; #: % of dry weight.

**Table 4:** Analytical methods of Gentaina species.

Method	Species	Parts	Source Herbs	Application	Sample Preparation	Details	Refs.
**Spectrometry**							
FT-IR	*G. macrophylla*, *G. crassicaulis*, *G. dahurica*, *G. straminea*	Roots	China	Investigate the different varieties and habitats plants by fingerprint analysis.	2 mg sample was blended with 200 mg KBr powder, ground again and pressed into a tablet.	Carried out on an FTIR spectrometer model IR200 and collected in the range of 4000–400 cm^−1^.	[139]
FT-IR	*G*. *crassicaulis*, *G*. *officinalis*, *G. straminea*, *G*. *lawrencei*, *G. rigescens*, *G. rhodantha*	/	China	Determine spectral differences among the selected species	Samples were pressed into thin tablets by tablet press.	Carried out on an FT-IR spectrometer (Perkin Elmer, Foster City, CA, USA) equipped with a DTGS detector and collected in the range of 4000–400 cm^−1^.	[50]
FT-IR	*G. rigescens*	Tissue culture of leaves	China	Metabolite variation in different plant parts and growing stages	2 mg sample was blended with 200 mg·KBr powder and pressed into a tablet.	Carried out on a Perkin-Elmer spectrometer equipped with a DTGS detector and collected in the range of 4000–400 cm^−1^	[107]
FT-IR	*G. rigescens*	Rhizomes	China	Analysis of different geographical origins	2 mg sample was blended with 200 mg KBr powder and pressed into a tablet.	Carried out on a Thermo Scientific Nicolet IR 100 FTIR spectrometer equipped with a deuterated triglycine sulfate detector, collected in range of 4000–400 cm^–1^.	[139]
NIR	*G. scabra*	Tissue culture of Herbs	Taiwan (China)	Determination of gentiopicroside	Powder was poured gently into a small ring cup (internal diameter (i.d.) 5 cm) and detected.	Carried out on NIRS 6500, FOSS NIR Systems and reflectance spectra were collected in the range of 400–249 nm with 2 nm interval.	[67]
FT-MIR	Raw and processed *G. rigescens*	Roots and Rhizome	China	Fingerprint analysis	2 mg sample was blended with 200 mg KBr powder, ground again and pressed into a tablet.	Carried out on a PerkinElmer spectrometer equipped with a DTGS detector and collected in the range of 4000–400 cm^−1^	[140]
**qHNMR**							
qHNMR	*Gentiana*e radix *G. scabrae* radix	Herbs	Japan	Determination of gentiopicroside	1. Ethanol extraction (UE, three times, 30 min, RT) then was evaporated; 2. Eluting with CHCl_3_:MeOH:H_2_O = 6:4:1; Fraction 3 was purified by SGCC (CHCl_3_:MeOH = 20:1→6:1) and ODS column chromatography (H_2_O:MeOH = 10:1→8:1) to yield gentiopicroside	Carried out on a JEOL JMN-ECA500 (500 MHz) spectrometer operating at 599.90 MHz equipped a PFG unit and a carousel autosampler	[141]
qHNMR	/	/	/	Determination of sweroside, swertiamarin, genipin, gentiopicroside, geniposide	/	Carried out on a Bruker Avance-400 NMR spectrometer operating at 400.13 MHz equipped with a 5 mm BBO probe head.	[142]
**TLC**							
HPTLC (280 nm)	*G. lutea*	Roots	France	Quantitative analysis of gentiopicroside.	Ethanol extraction (UE)	Solvent system: 1,2-dichloroethane–methanol–water(39:10:1), silica gel 60(F254).	[68]
HPTLC (270 nm)	*G. lutea*, *G. scabra*, Radix	commodity	Japan	Determination of gentiopicroside.	Ethanol extraction (UE)	Solvent system: CHCl_3_:MeOH:H_2_O = 30:10:1, Wakogel B-5fm plate.	[71]
HPTLC (270 nm)	*G. macrophylla*, *G. dahuricae*	commodity	China	Determination of gentiopicroside.	Ethanol extraction (UE)	Solvent system: CHCl_3_:MeOH:H_2_O = 30:10:1, Wakogel B-5fm plate.	[70]
HPTLC (270 nm)	*G. triflora var*. *japonica*, *G. scabra var*. *buergeri*	Leaves, Stems, Flowers, Roots	Japan	Determination of gentiopicroside	Ethanol extraction (UE)	Solvent system: CHCl_3_:MeOH:H_2_O = 30:10:1, Wakogel B-5fm plate.	[49]
**CE**							
CE-UV (254 nm)	*G. rigescens*	subterranean parts	China	Separation and determination of gentiopicroside and swertiamarin	Water extraction; (UE, twice times, 20 min)	CE-mode: MEKC-UV, 28 ± 1 °C, 15 kV. Detection separation conditions: 10 mM borax + 100 mM sodium dodecylsulfate (SDS) + ethanol at PH 8.00 Capillary	[142]
CE-UV (254 nm)	*G. rhodantha*, *G. kitag*, *G. scabra*, *G. rigescens*, *G. macrophylla*	/	China	Separation and determination of gentiopicroside and swertiamarin	1. Ethanol extraction (UE, 30 min); 2. The extracts were water extracted.	CE-mode: MEKC-UV, 15 kV. Detection separation conditions: 70 mM borate + 10 mM SDS + 6% (*v*/*v*) ispropanol (PH 9.0). Capillary: 50.0 cm (42.4 cm to the detector) × 75 µm i.d.	[75]
CZE-UV (270 nm)	*G. piasezkii*	whole plant	China	Quantitative analysis of luteolin, isoorient, 6′-*O*-vanilloylarbutin, lutonarin, 7-*O*-feruloylorientin.	1. 95% methanol extraction. (30 min, RT); 2. The extracts were water extracted then extracted with *n*-BuOH	CE-mode: CZE-UV, 25 °C,15 kV. Detection separation conditions: 200 mM boric acid buffer + 10% (*v*/*v*) methanol (PH 9.50) Capillary: Total length of 35 cm and effective length of 30 cm (50 μm I.D., 365 μm O.D.)	[86]
CE-DAD (260 nm for gentisin, isogentisin and 242 nm for amarogentin)	*G. lutea*	Roots	Austrian, German	Determination of gentisin, isogentisin, amarogentin	1. Ethanol extraction (UE, three times, 10 min, RT)	CE-mode: MEKC-DAD, 30 °C, 25 kV. Detection separation conditions: 100 mM sodium tetraborate buffer of (PH 9.3) +10 mM β-cyclodextrin. Capillary: 50 μm i.d. and effective length 60 cm.	[90]
CE-MS	*G*. *triflora*	Shoot tips	/	Metabolite profile anaylsis	1. Frozen material was ground to a powder in liquid nitrogen; 2. The powder was extracted with ice-cold 50% (*v*/*v*) methanol containing 50 µM methionine sulphone and 50 µM 1,4-piper-azinediethanesulfonic acid as an internal standard (4 °C, 5 min) after centrifugation; 3. The supernatant was filtrated before CE-MS analysis.	Running buffer: 20 mM ammonium acetate (PH 9.0) Capillary: polyethylene glycol-coated capillary	[143]
**GC**							
GC-FID	*G. lutea*	Roots	France	102 components identified	1. HD (2 h); 2. The oil separated on a silicagel column (20 cm × 1 cm) and eluted with *n*-hexane, *n*-hexane/CHCl_3_ (1:1) and CHCl_3_.	Capillary: Carbowax 20 M (50 m × 0.3 mm × 0.3 μm). Gradient temperature: 60 °C (3 min) to 180 °C (15 min), at 3 °C/min. Injection: Splitter.	[109]
GC-EI/MS	*G. lutea ssp aurantiaca Lainz* and *G. burseri Lap*	Roots, Rhizomes	Spain	83 compounds identified	1. SDE (40 min); 2. Volatile compounds were collected in about pentane; 3. Separated on a silica gel 60 column (70 × 4 mm i.d.), eluted with pentane–diethyl ether 98:2, 94:6 and 90:10 (*v*/*v*), and diethyl ether.	Capillary: HP-1 (50 m × 0.2 mm × 0.11 μm). Gradient temperature: 65 °C (4 min) to 240 °C (15 min), at 4 °C/min. Injection:.	[9]
GC-FID						Capillary: HP-19091Y-015(50 m × 0.32 mm × 0.30 μm). Other conditions: Described above GC-MS analysis	
GC-FTIR						Gradient temperature: 40 °C to 150 °C at 4 °C/min and from 150 °C to 240 °C. (15 min) at 10 °C/min. Other conditions: described above GC-MS analysis	
GC-FPD						Described above GC-MS analysis	
GC-olfactometry						Olfactometer on a 6 ft stainless steel column (1/8-in. o. d.) filled with methyl silicone. Other conditions: described above GC-MS analysis	
GC-EI/MS	*G. lutea*	Roots	Northwest of Spain	39 compounds identified	1. SE (150 rpm in a TOI); 2. Solvents: 50% ethanol, 50% propylene glycol ethanol, propylene glycol; 3. Etracts was SDE with 5% ethanol (40 min); 4. Volatile compounds were collected in about 20 mL of pentane.	Capillary: HP-1 (50 m × 0.2 mm × 0.11 μm) Gradient temperature: 65 °C (4 min) to 240 °C (15 min), at 4 °C/min. injection: no mention.	[116]
GC-FID						Capillary: HP-101 (50 m × 0.32 mm × 0.30 μm). Other conditions: described above.	
GC-EI/MS	*G. lutea*, *G. punctata*, *G. asclepiadea*	Flowers and Leaves	Reznjovete	81 compounds identified	1. HD (4 h); 2. The volatiles were collected in diethyl ether and dried over anhydrous sodium sulfate.	Capillary: HP5-MS (30 m × 0.25 mm × 0.25 μm) Gradient temperature: 40 °C to 280 °C, at 6 °C/min Injection: no mention.	[108]
GC-EI/MS	*G. triflora*, *G. scabra*, *G*. *triflora*×, *G. pneumonanthe*, *G. scabra*×, *G. pneumonanthe*, (*G. triflora*×, unknown)× unknown	Cultivar of Flowers	Japan	98 compounds were detected	HS-SPME 1. Fiber: PDMS and DVB/CAR/PDMS; 2. Extraction: 30 °C for 60 min; 3. Desorption: 230 °C for 5 min	Capillary: DB-WAX (60 m × 0.25 mm × 0.25 mm) Gradient temperature: 40 °C to 210 °C at 5 °C/min and maintained at 210 °C for 30 min Injection: HS-SPME	[110]
GC-FID	*G. kurroo*	Aerial parts	India	16 compounds were detected	HS-SPME 1. Fiber: carboxen-PDMS; 2. Extraction: 60 °C for 20 min; 3. Desorption: 250 °C for 5 min	Capillary: Fused silica capillary column (30 m × 0.32 mm × 0.25 µm) Gradient temperature: 60 °C to 280 °C injection: HS-SPME	[112]
GC-EI/MS						Capillary: VF-MS (60 m × 0.25 mm × 0.25 µm) Gradient temperature: 60 °C to 280 °C at 3 °C/min Injection: HS-SPME	
GC-EI/MS	*G. asclepiadea*	Root and Rhizome	Serbia	140 constituents were identified	1. HD (3 h)	Capillary: HP-5MS (30 m × 0.25 mm × 0.25 µm) Gradient temperature: 60 °C (for 5 min) to 130 °C (for 10 min) at 4 °C/min and then to 240 °C at 4 °C/min.	[29]
GC-FID		Aerial parts		90 constituents were identified			
GC-EI/MS	*G. nubigenan*	Flowers	china	71 components were identified	1. HRE (6 h)	Capillary: HP-5 (30 m × 0.25 mm × 0.25 µm) Gradient temperature: 80 °C to 290 °C (for 30 min) at 4 °C/min Injection: /	[88]
GC-EI/MS	*G*. *lutea*	Roots	Italy	154 compounds were identified	HS-SPME 1. Fiber: DVB/CAR/PDMS, PDMS/DVB, PDMS; 2. Extraction: 80 °C for 15 min; 3. Desorption: 250 °C for 3 min	Capillary: HP-5 (30 m × 0.25 mm × 0.1 µm) Gradient temperature: 50 °C(for 4 min) to 320 °C(for 2 min) at 4 °C/min Injection: HS-SPME	[111]
GC-EI/MS	*G. triflora*	Stems, Leaves	/	Identification of endogenous gibberellins	1. Endogenous gibberellins were extracted and purified by using several chromatographic steps.	Capillary: DB-1 fused silica capillary column (15 m × 0.25 mm × 0.25 µm) Gradient temperature: no mention Injection: /	[144]
**HPLC**							
HPLC (254 nm)	*G. macrophylla*, *G. crassicaulis*	Roots	China	Simultaneous analysis loganic acid, sweroside, gentiopicroside, swertiamarin	70% methanol extraction (UAE)	Mobile phase: A: acetonitrile, B: 1% aqueous acetic acid. 0–25 min: 5–10% A, 25–45 min: 10–45% A, 45–55 min: 100% A, 55–65 min: 5% A. Flow rate: 1 mL/min Column: Agilent Extend-C18 (250 mm × 4.6 mm, 5 μm)	[56]
HPLC-UV (254 nm)	*G. pannonica*, *G. punctata*, *G. purpurea*	Herbs	European	Determination of amarogentin, amaropanin	Methanol extraction	Mobile phase: A:methanl-B: phospbatc buffer(0.01 M, PH 5). 0–5 min: 10% A to 25% A, 5–20 min: 25% A Flow rate:1 mL/min Temperature: 60 °C Column: ODS-HG SIL-X-1 (250 mm× 2.6 mm) (Perkin-Elmer)	[79]
HPLC-UV (275 nm)	*G. lutea*	Protoplasts and vacuoles of Roots	Germany	Analysis of gentiopicroside	90% methanol extraction	Mobile phase: methanol:water = 55:45 Flow rate: 1 mL/min Column: ODS-Hypersil-5 μm (Hyperchrome, 250 mm × 4.6 mm)	[64]
HPLC-UV (254 nm)	*G. lutea*	Roots	Turkey	Analysis of extracts (swertiamarin, gentiopicrin, sweroside)	Methanol extraction (Soxhlet, 4 h)	Mobile phase: Methanol/water (30:70 *v*/*v*) Flow rate: 1 R/min Column: Ultracarb ODS-C20 (150 mm × 4.6 mm i.d. 5 μm) (Phenomenex, Torrance, CA, USA)	[72]
HPLC-UV (354 nm)	*G. olivieri*	Aerial	Turkey	Analysis of isoorientin	1. 80 ethanol extraction (soak, + stirring, 3 h, RT); 2. The extracts was dissolved in water and fractionated through successive extractions with chloroform, ethyl acetate, n-butanol/saturated with water.	Mobile phase: Water:Methanol:anhydrous acetic acid (65:35:5) Flow rate: 0.8 mL/min Column: LiChrospher RP-18 (250 mm × 4.6 mm, i.d. 5 μm)	[82]
HPLC-UV (354 nm)	*G. olivieri*	Aerial	Turkey	Determination of isoorientin	1. Water extraction ( stirring, 40 °C, 24 h, two times) then was evaporated; 2. The residues was dissolved and diluted in methanol. Methanolic extracts through successive solvent extractions as follows: ethyl acetate, *n*-butanol, aqueous, methanol-Water.	Mobile phase: Water:Methanol:Glacial acetic acid = (65:35:5) Flow rate: 0.8 mL/min Column: Lichrosphere RP-18 (250 mm × 4.6 mm i.d.5 μm)	[21]
HPLC-UV (254 nm)	*G. straminea*	Roots, Rhizomes Leaves	China	Determination of gentiopicroside contents of different habitats, origans and harvest time	Methanol extraction. (HRE, 30 min)	Mobile phase: Water:Methanol = 3:1 Flow rate: 1.1 mL/min Temperature: 30 °C Column: ODS	[61]
HPLC-UV (254 nm)	Endophytic fungal strain isolated from *G. macrophylla*	Roots, Rhizomes Leaves	/	Determination of gentiopicroside	Methanol extraction	Mobile phase: methanol:water = 1:4 (*v*/*v*) Flow rate: 1 mL/min Temperature: 25° Column: YWG-C18 column (150 mm × 4.6 mm)	[115]
HPLC–UV (240 nm)	*G. straminea*, *G. dahurica*, *G. crassicaulis*, *G. waltonii*, *G. officinalis*, *G*. *ihassica*, *G. macrophylla*	Roots	China	Determination of loganic acid, sweroside, gentiopicroside, swertiamarin	Methanol extraction (60 °C, 3 min)	Mobile phase: MeOH-0.5% AcOH-aqueous. 0–10 min: 19% A, 10–20 min: 25% A. Flow rate: 1.0 mL/min Column: Alltech C18 (250 mm × 4.6 mm, 5 μm)	[44]
HPLC-UV (240 nm)	*G. macrophylla*	Flowers Roots	China	Detect changes in sweroside, longanic acid, gentiopicroside, and swertiamarin	50% Methanol extraction (UAE)	Mobile phase: A: 0.3% formic acid water, B: methanol. Flow rate: 1 mL/min Temperature: RT Column: Agilent Eclipse Plus C18 (250 mm × 4.6 mm, 5 μm)	[99]
HPLC-UV	*G. lutea*	Leaves and roots	Slovenia	Determination of mangiferin, amarogentin, isoorientin, gentiopicroside, isovitexin	Ethanol or (0%, 20%, 40%, 60%, 80%, 100%) methanol extraction (UE, 15 min)	Mobile phase: A: acetonitrile, B: 0.1 N H_3_PO_4_ in water. 0–5 min: B: 98%, 5–18 min: B: 90%, 20–25 min: B: 85%, 30 min: B: 70%, 40 min: B: 30%, 50 min: B: 0%. Flow rate: 0.8 mL/min Column: Lichrospher RP18 (250 mm × 4 mm, i.d. 5 μm)	[15]
HPLC-UV (242 nm)	*G. macrophylla*, *G. straminea*, *G*. *dahurica*, *G. crassicaulis*, *G*. *officinalis*, *G. siphonantha*	Herbal	China	Simultaneous determination of loganic acid, swertiamarin, gentiopicroside, sweroside and fingerprint analysis	Methanol extraction (UE, 40 min).	Mobile phase: A: 0.4% aqueous phosphoric acid, B: methanol. 0–40 min, linear 10–30% B. Flow rate: 1.0 mL/min Temperature: 25 °C Column: C18 column (Phenomenex, 150 mm × 4.6 mm, 5 μm)	[45]
HPLC-UV (354 nm)	*G. olivieri*, *G. asclepiadea*, *G. cruciate*, *G. gelida*, *G*. *septemfida*	Flowers, leaves, and stem	Turkey	Quantitative determination of isoorientin.	Methanol extraction (twice times, 40 °C, 24 h)	Mobile phase: water-methanol-glacial acetic acid (65:35:5, *v*/*v*/*v*) Flow rate: 0.8 mL/min Temperature: 30 °C Column: LiChrospher 100 RP-18 (5 μm, 4.6 mm × 250 mm)	[82]
HPLC-UV (242 nm)	*G. macrophylla*, *G. straminea*, *G. crassicaulis*, *G. dahurica*	Roots	China	Determination of gentiopicroside and genetic diversity.	Methanol extraction (UE, 30 min) and water extraction	Mobile phase: A: 0.4% aqueous phosphoric acid, B: methanol. 0–40 min, linear 10–30% B. Flow rate: 1.0 mL/min Temperature: 25 °C Column: C18 column (Phenomenex, 150 mm × 4.6 mm, 5 μm)	[145]
HPLC-UV (210 nm)	*G. olivieri*	Fowers	India	Quantiication of oleanolic acid	Methanol extraction	Mobile phase: A: methanol, B: 0.03 mol/L phosphate buffer (PH 2.9). A:B = 85:15 (*v*/*v*) Flow rate: 0.3 mL/min Temperature: 20 °C Column: ODS C18 (250 mm × 4.6 mm, 5 μm).	[87]
HPLC-PDA (240 nm)	*G*. *scabra*, *G. arisanensis*, *G. davidii var*. *formosana*, *G. scabrida var*. *punctulata*	Roots	Taiwan (China)	Determination of swertiamarin and gentiopicroside	Methanol extraction (UE, 40 °C, 30 min, three times).	Mobile phase: A: 0.2% phosphoric acid in water, B: methanol. 0–25 min: 80–65% A, 25–30 min: 80% A. Flow rate: 1.0 mL/min Temperature: 25 °C Column: Mightysil RP-18 GP (250 mm × 4.6 mm, 5 μm).	[54]
HPLC-DAD (232 nm)	*G. cruciata*	Hairy roots of Roots	/	Determined the secondary metabolite content (loganic acid, swertiamarin, gentiopicroside, sweroside, amarogentin, gentioside, gentisine, isogentisin)	Methanol extraction (times with)	Mobile phase: A: 0.025% of trifluoroacetic acid in water, B: acetonitrile:*n*-propanol = (1:1). 0–20 min: A: 99% to 70%, 20–20.5 min: A: 70% to 60%, 20.5–30 min: A: 60%, 30–30.5 min: A: 60% to 5%, 30.5–35 min: A: 5%. Flow rate: 1.0 mL/min Temperature: 30 °C Column: Zorbax Eclipse XDB-C18 (150 mm × 4.6 mm, 5 µm)	[58]
HPLC-DAD (230 nm)	*G. cruciata*	Clones of Roots	/	Determined the secondary metabolite content (loganic acid, swertiamarin, amarogentin, sweroside, gentiopicroside, gentisine, isogentisin, gentioside, gentioside-isomer).	Methanol extraction (twice times, RT, 24 h).	Mobile phase: A: 0.025% of trifluoroacetic acid in water, B: acetonitrile:*n*-propanol = 1:1. 0–20 min: A: 99% to 70%, 20–20.5 min: A: 70% to 60%, 20.5–30 min: A: 60%, 30–30.5 min: A: 60% to 5%, 30.5–35 min: A: 5%. Flow rate: 1 mL/min Temperature: 30 °C Column: XDB-C18 column (4.6 mm × 250 mm, 5 μm)	[55]
HPLC-PDA (350)	*G. piasezkii*	Whole plants	China	Simultaneous determination of flavonoids (lutonarin, saponarin, luteolin, isoorientin, 7-*O*-feruloylorientin)	1. 80% ethanol extraction then water, CHCl_3_, *n*-BuOH extraction (three times) to obtain the *n*-BuOH extracts; 2. The *n*-BuOH extracts were evaporated and dissolved in methanol	Mobile phase: A: acetonitrile, B: water–acetic acid (99:1, *v*/*v*). 0–4 min: 16% A, 4–20 min: 40% A. Flow rate: 0.8 mL/min Temperature: 25 °C Column: Kromasil C18 column (5 μm, 4.6 mm × 250 mm).	[84]
HPLC-PDA (240 nm, 254 nm)	*G. farreri*, *G. sino-ornata* and their hybrid, *G. “Macazdayi”*	Leaves and Stems	Switzerland	Analysis of secoiridoid glycosides and flavonoid glycosides (swertiamarin, isoscoparin, gentiopicroside, sweroside, isoorientin, isoorientin-4′-*O*-β-d-glucoside)	1. Light petroleum (80–95 °C), CHCl_3_ and methanol sequential extraction; 2. Methanolic extracts by column chromatography on Polyamide SC6 with 20% and 90% aqueous methanol to obtain secoiridoid and the flavonoid fractions, respectively, for HPLC	Secoiridoid analysis: Mobile phase: 20% methanol water Flow rate: 1.2 mL/min Flavonoid analysis: Mobile phase: A: 0.03% phosphoric acid water-B: methanol. 0–15 min: 20% A to 50% A Flow rate: 1.5 mL/min Column: Hypersil RP-8 (100 mm × 4.6 mm, i.d. 5 μm, Hewlett-Packard)	[73]
HPLC-DAD (233 nm, 270 nm)	*G. lutea*	Roots	Northwest Spain	Determination of amarogentin and gentiopicroside	1. SE (150 rpm in a TOI); 2. Solvents: 50% ethanol, 50% propylene glycol ethanol, propylene glycol.	Amarogentin analysis: Mobile phase: methanol:water = 43:57 (*v*/*v*) Gentiopicroside analysis: Mobile phase: methanol:water = 30:70 (*v*/*v*), Flow rate: 1 mL/min Column: LiChrosorb RP18 (250 mm × 4 mm i.d., 10 μm)	[116]
HPLC-DAD	*G. lutea*	Leaves and Flowers	Suvobor, Stara Planina, Koritnik, Ošljak.	Analysis of compounds and seasonal variations (swertiamarin, gentiopicrin, mangiferin, isoorientin, isovitexin, isogentisin, isogentisin-3-*O*-β-d-primeveroside.)	Methanol extraction (HRE, 30 min)	Mobile phase: A: acetonitrile-B: 0.1 NH_3_PO_4_ in water. 0–5 min: B: 98%, 5–18 min: B: 90%, 20–25 min: B: 85%, 30 min: B: 70%, 40 min: B: 30%, 50 min: B: 0% Flow rate: 0.8 mL/min Column: Lichrospher RP18 (250 mm × 4 mm, i.d. 5 μm)	[16]
HPLC-DAD-ESI-MSD (233 nm)	*G. cruciata*, *G. pneumonanthe*, *G. ascjepiadea*, *G*. *lutea*	Roots and Rhizomes	Hungary	Qualitative (method A) and quantitative (method B) analysis of compounds (gentiopicroside, swertiamarin, sweroside, gentisine, amarogentin, gentioside-isomers)	Methanol extraction (SE)	Method A: Mobile phase: A: methanol-B: water. 0–30 min: A: 10% to 100%, 30–37 min: A: 100% Flow rate: 1 mL/min Temperature: 40 °C MS: negative ion mode Column: Eurospher-100 C18 (250 mm × 4 mm) Method B: Mobile phase: A: methanol-B: water. 0–6 min: A: 16% to 30%, 6–9 min: A: 30% to 100%, 9–10.5 min: A: 100% Flow rate: 1 mL/min Temperature: 40 °C Column: Eurospher-100 C18 (150 mm × 4 mm)	[76]
HPLC-PDA (239 nm)	*G*. *lutea*	Roots	France	Determined iridoid and xanthone compounds (gentiopicroside, swertiamarin, loganic acid, gentisin, isogentisin, gentioside, 1-hydroxy-3-methoxy-7-*O*-primeverosyl xanthone)	Methanol extraction (HRE, 30 min)	Mobile phase: A: water/phosphoric acid 85% (100:0.3 *v*/*v*). B:acetonitrile/water/phosphoric acid 85% (80:20:0.3 *v*/*v*/*v*). 0–15 min: B: 10–20%, 15–30 min: B: 20–45%, 30–40 min: B: 45–50%, 40–45 min: B: 50% Flow rate: 2 mL/min Column: Lichrocart125-4 Superspher RP8-E 5 µm (Merck, Darmstadt, Germany)	[51]
HPLC-PDA	*G. manshurica*, *G. scabra*, *G. triflora*, *G. rigescens*, *G. rhodantha*, Radix and adulterant: Podophyllum hexandrum Royle	Roots, Rhizomes	China	Isolation of secoiridoid glycoside (gentiotrifloroside, loganic acid, sweroside, swertiamarin, gentiopicroside, 6′-*O*-β-d-glucopyranosyl-gentiopicroside, 2′-(o,m-dihydroxybenzyl)-sweroside)	Methanol extraction (UE)	Mobile phase: A: water B: acetonitrile. 0–22.5 min: 10% of B, 22.5–25 min: 10–20% of B, 25–32.5 min: 20% B, 32.5–35 min: 20–10% of B. Column: C18 column (250 mm × 4.6 mm, 5 μm; Beckman, Brea, CA, USA)	[66]
HPLC-DAD (260, 320 nm)	*G. lutea*	Flowers, Leaves	Serbia	Investigate the antimicrobial activity and quantification of secondary metabolites (mangiferin, isogentisin gentiopicroside)	Methanol extraction (Soxhlet, 24 h)	Mobile phase: A: 1% H_3_PO_4_(0.1N)H2O, B: acetonitrile. 0–5 min: 98–90% A, 5–10 min: 90% A, 10–13 min: 90–85% A, 13–15 min: 85% A, 15–20 min: 85–70% A, 20–24 min: 70–40% A, 24–28 min: 40–0% A. Flow rate: 1 mL/min Column: Zorbax SB–C18 (150 mm × 4.6 mm i.d., 5 μm)	[62]
HPLC-DAD (260, 320 nm)	*G. dinarica*	Aerial roots (plants from in vitro culture and nature)	/	Analysis of secoiridoids and xanthones (gentioside, swertiamarin, gentiopicroside, and norswertianin-1-*O*-primeveroside)	Methanol extraction (48 h, RT).	Mobile phase: A: H_2_O containing 1% 0.1 NH_3_PO_4_, B: acetonitrile. 0–5 min: 98–90% A, 5–10 min: 90% A, 10–13 min: 90–85% A, 13–15 min: 85% A, 15–20 min: 85–70% A, 20–24 min: 70–40% A, 24–28 min: n 40–0% A. Flow rate: 1.0 mL/min. Column: Zorbax SB–C18 (150 mm × 4.6 mm, 5 μm)	[65]
HPLC-DAD (238 nm)	*G. rigescens*, *G. scabra*	Herbs	China	Fingerprint analysis and quantitative analysis of loganic acid, swertiamarin, gentiopicroside, sweroside	50% methanol extraction (UE, 60 min, 60 °C)	Mobile phase: A: methanol, B: 0.1% phosphoric acid. 0–4 min, 25%A, 4–10 min, 25–35% A, 10–20 min, 35–40% A. Flow rate: 1.0 mL/min Temperature: 25 °C Column: Zorbax SB C18 (4.6 mm × 250 mm, 5 μm)	[46]
HPLC-DAD (254 nm)	*G. straminea*	Somatic embryogenesis	/	Determined gentiopicroside	Methanol extraction (1 h, RT).	Mobile phase: water—methanol (80:20) Flow rate: 1.5 min/mL Column: RP–C18 (4.6 mm × 150 mm, 5 μm)	[127]
HPLC-FLD-APCI-MS (excitation and emission wavelengths were 260 and 380 nm)	*G. straminea*, *G. dahurica*	Roots	China	Determination of free fatty acids	1. CHCl_3_ extraction (UE, 20 min, three times); 2. Extracts and Pyridine were mixed and ultrasonicated for 20 s then evaporated; 3. The residue was dissolved in HPLC-grade DMF and derivatized. Precolumn derivatization: TSPP as a fluorescence reagent, K_2_CO_3_ as catalyst, DMF as solvent, temperature 90 °C. diluted solution: aqueous:acetonitrile (1:1 *v*/*v*)	Mobile phase: A: CH_3_CN–water (1:1 *v*/*v*), B: CH_3_CN–water containing 0.2 mol/L HCOONH_4_ buffer, C: CH_3_CN–DMF (100:2 *v*/*v*), D: CH_3_CN–DMF (100:30 *v*/*v*). 0–4 min: A: 95%, C: 5%, 4–4.2 min: B: 95%, C: 5%, 8 min: B: 95%, C: 5%, 8.5 min: B: 75%, C: 25%, 15 min: B: 50%, C: 50%, 40 min: C: 100%, 48–65 min: D: 100%. Flow rate: 1 mL/min MS: positive-ion mode Column: Eclipse XDB–C8 (4.6 mm × 150 mm, i.d. 5 μm)	[100]
HPLC-FLD-ESI/MS (excitation, emission wavelengths were 333 and 390 nm)	*G. dahurica*	Roots	China	Determination of free fatty acids	1. 80% ethanol macerated for 24 h, extracted (UE, 1 h, RT) and evaporated; 2. The residue was dissolved in water with borate buffer (0.2 mol/mL, 2.0 mL) and prepared for derivatization. Precolumn derivatization: 1. Extracts, acetonitrile, 0.2 mol/L borate buffer (PH 9.0) and BCEOC were mixed and carried out in a water bath (30–40 °C, 10 min); 2. The excess reagent was removed by hexane/ethyl acetate (10:1, *v*/*v*); 3. The aqueous phase was diluted by 36% formic acid to obtain a final pH: 6.0–6.5; 4. The derivatization solution was analyzed.	Mobile phase: A: 30% acetonitrile with 30 mmol/mL formic acid buffer (PH = 3.7), B: 50% acetonitrile, C: 95% acetonitrile. 0 min: A: 70%, B: 30%, 15 min: A: 55%, B: 45%, 20 min: A: 2%, B: 98%, 28 min: A: 2%, B: 96%, C: 2%, 28.3 min: A: 2%, B: 88%, C: 10%, 30 min: A: 2%, B: 83%, C: 15%, 40 min: B: 80%, C: 20%, 50 min: B: 20%, C: 80%, 55 min: B: 5%, C: 95%, 57–65 min: C: 100%. Flow rate: 1.0 mL/min Temperature: 30 °C Column: Hypersil BDS C18 (4.6 mm × 200 mm; 5.0 μm, Yilite Dalian, China)	[101]
HPLC-ELSD	*G. dahurica*	Roots	China	Determination of acid hydrolysate of 6′-*O*-Acetyl-gentiopicroside and 3′-*O*-Acetyl-gentiopicroside	1. Compounds were obtained through ethanol extraction; 2. The compounds were added to HCl and heated (85 °C, 12 h); 3. The mixture was diluted with H_2_O and extracted with ethyl acetate (three times) and neutralized with NaHCO_3_	Mobile phase: H_2_O Flow rate: 0.2 mL/min Temperature: 25 °C Column: Waters Sugar–PakTM 1 (300 mm × 6.5 mm)	[77]
HPLC-PDA (232 nm)	*G. lutea*	Roots	Austria	Simultaneous determination of loganic acid, swertiamarin, gentiopicroside, amarogentin, gentioside gentioside-isomer, gentisin, isogentisin	Methanol extraction (UE, 10 min, RT)	Mobile phase: A: 0.025% of TFA in water B:acetonitrile:*n*-propanol (1:1). 0 min: A: 99%, B: 1%, 20 min: A: 70%, B: 30%, 20.5–30 min: A: 60%, B: 40%, 30.5–35 min: A: 5%, B: 95%. Flow rate: 1 mL/min Temperature: 30 °C Column: Zorbax Eclipse XDB–C18 column (150 mm × 4.6 mm, i.d. 5 μm)	[48]
HPLC-ESI/MS						Mobile phase: A: water:formic acid:acetic acid (99:0.9:0.1), B:acetonitrile:*n*-propanol (1:1). 0 min: A: 99%, B: 1%, 20 min: A: 70%, B: 30%, 20.5–30 min: A: 60%, B: 40%, 30.5–35 min: A: 5%, B: 95%. Flow rate: 1 mL/min MS: negative-ion mode Temperature: 30 °C Column: Zorbax Eclipse XDB-C18 column (150 mm × 4.6 mm, i.d.5 μm)	
HPLC-PDA (254 nm)	G. *lutea*	Plant material and root	Germany and Austria	Isolation and simultaneous determination of bioactive compounds (loganic acid, swertiamarin, sweroside, gentiopicroside, amarogentin, 1,6,8-triOH-3,5,7-triMeO-xanthone).	Methanol extraction	Mobile phase: A: 0.085% (*v*/*v*) H_3_PO_4_, B: acetonitrile. 0 min: A: 95%, 12 min: A: 80%, 20 min: A: 70%, 40 min: A: 20%, 45 min: A: 2%, 53 min: A: 2%, 53.5 min: A: 95%, 65 min: A: 95%. Flow rate: 1.0 mL/min Temperature: 40 °C Column: Zorbax Eclipse XDB-C18 (150 mm × 4.6 mm, 5μm)	[54]
HPLC-ESI/MS						Mobile phase: A: water:formic acid = 99.9:0.1 (*v*/*v*), B: acetonitrile. other condition described above MS: positive ESI mode	
HPLC-UV (270 nm)	*G. rigescens*	Roots	China	Fingerprint analysis of the purity of polysaccharide (named RG4-1)	1. Water extraction (100 °C, 1 h), then precipitated by 90% ethanol (48 °C overnight); 2. The concentrated crude polysaccharides were eluted with deionized water, followed by 30%, 60%, and 90% ethanol; 3. The water-eluted part was eluted with deionized water; 4. The first part of the fraction was named RG4-1; 5. RG4-1 was dissolved in deionized water and injected into HPLC.	Mobile phase: Methanol:water = 70:30 Flow rate: 1.0 mL/min Temperature: 25 °C Column: C18 (4.6 mm × 250mm, 5 μm)	[95]
HPLC-RID				Analysis of the purity of polysaccharide (named RG4-1).		Mobile phase: isotonic NaCl Flow rate: 1.0 mL/min Temperature: 25 °C Column: Spherisorb ODS2 (125 mm × 4.0 mm, 5 μm)	
HPLC-UV (236 nm)	*G. macrophylla*	Roots	China	Fingerprint analysis (loganic acid, swertiamarin, gentiopicroside, sweroside)	Methanol extraction (30 min, 30 °C)	Mobile phase: A: CH_3_OH, B: 0.4% aqueous phosphoric acid. A:B = 25:75. Flow rate: 1.0 mL/min. Temperature: 30 °C. Column: Welchrom C18 (250 mm × 4.6 mm, 5 μm)	[52]
LC-UV-ESI-TOF/MS (236 nm)						Mobile phase: A: CH_3_OH, B: 0.1% aqueous formic acid. 0–28 min, 31% A, 28–29 min, 31–37% A, 29–40 min, 37% A. Flow rate: 0.3 mL/min. Temperature: 30 °C MS: positive ion mode Column: Welchrom C18 (250 mm × 4.6 mm, 5 μm)	
LC-UV-ESI/MS (242 nm)	*G. straminea*, *G*. *macrophylla*, *G. crassicaulis*	Roots, herbs	China	Simultaneous determination of loganic acid, swertiamarin, sweroside, gentiopicroside, 6′-*O*-acetyl-gentiopicroside 7(*R*)-*n*-butyl-morroniside, 7(*S*)-*n*-butyl-morroniside, 6′-*O*-β-d-glucosyl-gentiopicroside, 2′-*O*-(2,3-dihydroxyl- benzoyl)-sweroside, 6′-*O*-(2-hydroxyl-3-*O*-β-d-glucopyranosyl- benzoyl)-sweroside	Methanol extraction (1.5 h, RT, twice times)	Mobile phase: A: methanol, B: water with 0.04% phosphoric acid. 0–10 min, 10–20% A, 10–20 min, 20% A, 20–60 min, 20–75% A. Flow rate, 1.0 min/mL Temperature: 25 °C MS: positive ESI mode Column: Kromasil-C18 (200 mm × 4.6 mm, 5 μm)	[47]
HPLC-DAD-ESI/MS (260, 325 nm)	*G. asclepiadea*	Haulm and Flower	Slovakia	Identification of mangiferin, sweroside, gentiopicroside, swertiamarin, isovitexin, isoorientin)	1. Methanol or water extraction (65 °C, 5 times); 2. The extracts were filtered and concentrated (the water was removed by azeotropic distillation with benzene); 3. The residue was dissolved in methanol or water before analysis.	Mobile phase: A: methanol, B: 4.8% formic acid in water. 0–30 min: 15% A to 70% A. Flow rate: 1.0 mL/min MS: positive and negative ion mode Column: Zorbax XDB C8 (150 mm × 4.60 mm, 3.5 μm)	[78]
				Analyses of compounds swertiamarin, isovitexin, gentiopicroside, isoorientin, sweroside			[85]
HPLC-DAD-MS (254 nm)	*G. macrophylla*, *G. straminea*	Flower	China	Determined the chemical compounds (vitexin, loganic acid, swertamarin, sweroside, gentiopicroside, isoorientin-4′-*O*-β-d-glucoside, 6′-*O*-β-d-glucopyranosyl-gentiopicroside)	Methanol extraction (UE), evaporation and dilution with 10% methanol.	Mobile phase: A: 1% acetic acid, B: acetonitrile. 0–13 min: B: 95–90%, 13–48 min: B: 90–87.5%, 48–70 min: B: 87.5–80%, 70–75 min: B: 80%, 75–80 min: B: 80–95%, 80–90 min: B: 95%. Flow rate: 1.0 mL/min Temperature: 25 °C MS: positive and negative ion mode Column: Waters SunFireC18 (4.6 mm × 250 mm, 5 μm)	[40]
HPLC-DAD-ESI/MS (275 nm)	*G. veitchiorum*	Fowers	China	Analyses of gentiopicroside	Ligarine extraction (HRE, 30 min) then dried and extracted with methanol (UE, 40 min)	Mobile phase :A: water, B: 0.5% acetic acid in methanol. 0–6 min: 15–20% (B), 6–20 min: 20–40% (B), 20–30 min: 40–60% (B), 30–35 min: 60–35% (B). Flow rate: 1.0 mL/min Temperature: 40 °C MS: positive ion mode Column: RP C18(5 μm, 150 mm × 0.5 mm Agilent).	[62]
HPLC-DAD (260 nm)	*G. asclepiadea*	Root	Serbia	Determination of sweroside, swertiamarin, gentiopicroside, mangiferin.	Methanol extraction (RT, 24 h) then evaporated and sequential extraction through water, chloroform, ethyl acetate and *n*-butanol.	Mobile phase: A: acetonitrile, B: 0.2% phosphoric acid. 0–2 min: 100–98% B, 2–5 min: 98–90% B, 5–10 min: 90–80% B, 10–20 min: 80–0% B. Flow rate: 0.5 mL/min Column: Hypersil BDS-C18 (5 μm, 125 mm × 2 mm)	[80]
		Whole plant		Determination of sweroside, mangiferin, swertiamarin, gentiopicroside	Methanol extraction (24 h, RT, three times)		[103]
	G. *cruciata*	Aerial, Roots	East Serbia	Analysis of sweroside, mangiferin, swertiamarin, gentiopicroside			[81]
HPLC-DAD (254 nm)	*G. macrophylla*	Flowers	China	Analysis of loganic acid, swertamarin, gentiopicroside and sweroside	1. 75% ethanol extraction then evaporation and sequential extraction through water, petroleum ether, chloroform3, ethylc acetate and *n*-BuOH extraction; 2. The *n*-BuOH fraction was subjected to column chromatography (CHCl_3_/MeOH/H_2_O gradient (10:3:1–5:3:1)) and yielded the iridoid glycoside fraction	Mobile phase: A: 1‰ acetic acid, B: acetonitrile. 0–20 min, 95–90% (B); 21–40 min, 90–87.5% (B); 41–60 min, 87.5–80% (B); 61–90 min, 80% (B); 91–100 min, 80–95% (B). Flow rate: 0.8 mL/min, Temperature: 25 °C Column: Waters SunFire C18 (4.6 mm × 250 mm, 5 μm)	[59]
HPLC-PDA	*G. macrophylla*	Roots	Taiwan (China)	Analysis of compositions and contents of flavonoids and phenolic acids	80% ethanol extraction (soaked, 1 week)	Mobile phase: A: methanol, B: 9% glacial acetic acid solution. 0–5 min: 5% to 17% A, 5–25 min: 17% A, 25–40 min: 17% to 31% A, 40–76 min: 31% A, 76–80 min: 31–40% A, 80–120 min: 40% A. Flow rate: 0.8 mL/min Column: Hypersil GOLD C18 (250 × 4.6 mm, 5 μm)	[146]
HPLC–ESI/MS	*G*. *kurroo*	Roots	Kashmir Himalaya	11 main chemical constituents were identified	Methanol extraction (Soxhlet, 60–65 °C, 2–3 h).	Mobile phase: A: aqueous formic acid (0.1%), B: methanol. 0–8 min: 12% to 25% B, 12–16 min: 25% B, 16–40 min: 25% to 40% B, 16–40 min: 40% to 50% B, 40–50 min: 50% to 100% B. Flow rate: 0.5 mL/min MS: positive and negative ion mode Column: Chromolith RP-18 (4.6 mm ID, 50 mm length)	[105]
HPLC-DAD-ESI/MS	G. *triflora*	/	/	Analysis of structural characterization of 11 secoiridoids glycosides	Methanol extraction (UE, 30 min, three times)	Mobile phase: A: acetonitrile, B: water. 0–15 min, 10–40% A, 15–30 min,40–60% A, 30–40 min, 60–90% A, 40–41 min, 90–100%A, 41–50 min, 100% A. Flow rate: 0.6 mL/min Temperature: 25 °C MS: positive and negative ion mode Column: C18 (4.6 mm × 250 mm, 5 μm; Agilent, Santa Clara, CA, USA)	[147]
HPLC–DAD–ESI/MS (254 nm)	*G*. *rigescens*, *G. scabra*, *G. triflora*, *G*. *purdomii*, *G. erectosepal*a, *G. obconica*, *G. microdonta*, *G. crassicaulis*, *G. straminea*, *G. dahurica*, *G. macrophylla*, *G*. *robusta*	Roots	China	Fingerprint analysis and identification of compounds (loganic acid, sweroside, swertiamarin, gentiopicroside, 2′-(o,m-dihydroxy-benzyl)-sweroside).	Methanol extraction (soak, 8 h and UE, 30 min).	Mobile phase: A: 0.2% (*v*/*v*) H_3_PO_4_, B: acetonitrile. 0–25 min: B: 8–20%, 25–26 min: B: 20–100%. Flow rate: 1 mL/min Temperature: 40 °C MS: positive and negative ion mode Column: ZORBAX SBC18 (4.6 mm × 150 mm, 5 μm)	[23]
HPLC-DAD-ESI/MS (232, 246, 258, 275 nm)	*G. lutea*	Roots	Pian Perduto	Simultaneous analysis of loganic acid, sweroside, swertiamarin, amarogentin, gentiopicroside, isogentisin,	Methanol extraction (UE, 45 min, RT)	Mobile phase: A: 0.1% aqueous acetic acid, B: acetonitrile. 0–7 min, 20% B, 7–15 min, 20–90% B, 15–18 min, 90% B, 18–25 min, 90–20% B. Flow rate: 0.6 mL/min Temperature: 11 °C MS: negative ion mode Column: Synergi Polar-RP C18 (4.6 mm× 150 mm, 4 μm)	[11]
HPLC-ESI/MS	*G. scabra*	Roots	South Korea	Fingerprinting analysis (loganic acid, rindoside, gentiopicrin, trifloroside)	Water extraction (UE, 3 h, 40 °C, two times).	Mobile phase: A: 0.1% formic acid water, B: acetonitrile. 0–5 min: B: 5%, 5–60 min: B: 5–90%, 60–70 min B: 90%, 70–75min B: 90–5%. Flow rate: 50 μL/min Temperature: 40 °C MS: positive and negative ion mode Column: Luna C18 (150 mm × 1.0 mm i.d., 5 μm, Phenomenex, Torrance, CA, USA)	[57]
Microcolumn-HPLC-UV-ESI/MS (254 nm,334 nm)	*G. decumbens*, *G. macrophylla*, *G. triflora*, *G*. *algida*	Herbs	Siberian	Determination of loganic acid, swertiamarin, gentiopicroside, sweroside, isosaponarin, mangiferin, saponarin, isoorientin, isovitexin, isoscoparin, loganic acid-6′-*O*-d-glucoside, isoorientin-4′-*O*-β-d-glucoside.	Methanol extraction UE, 40 min, 70 °C)	Mobile phase: A: 0.2 М LiClO4 in 0.006M HClO4, B: acetonitrile. 0–2.5 min: 5–35% B, 2.5–4 min, 35–70% B. Flow rate: 600 μL/min. Temperature: 35 °C Column: ProntoSIL-120-5-C18AQ (1 mm × 50 mm, 1 μm)	[30]
UPLC-ESI/MS (242 nm)	*G. crassicaulis*, *G. officinalis*, *G. straminea*, *G. lawrencei*, *G*. *rigescens*, *G*. *rhodantha*	/	China	Fingerprinting and quantitative analysis of loganic acid, swertiamarin, gentiopicroside, sweroside, mangiferin, isoorientin, isovitexin.	1. Methanol extraction (UE, 35 min)	Mobile phase: A: 0.1% formic acid in water, B: acetonitrile. 0–0.30 min: 6% B, 0.31–13.70 min: 6–15.5% B, 13.71–21.20 min: 15.5–38% B, 21.21–27.00 min: 38–83% B. Flow rate: 0.35 mL/min. Temperature: 45 °C MS: positive and negative ion mode Column: Shim-pack XR–ODS III (150 mm × 2.0 mm, 2.2 μm)	[50]
UFLC-UV-MS/MS (242 nm)	*G. rigescens*	Seed, Root, Stem, Leaf Flower	China	Fingerprinting and quantitative analysis (loganic acid, sweroside, swertiamarin, gentiopicroside)	Methanol extraction (UE, 35 min)	Mobile phase: A: 0.1% formic acid in water, B: methanol. 0–0.30 min: 13% B, 0.31–7.00 min: 13%–20% B, 7.01–13.00 min: 20–46% B, 13.01–16.50 min: 46–60% B 16.51–19.00 min: 60–90% B. Temperature: 45 °C. Flow rate: 0.4 mL/min MS: positive and negative ion mode Column: Shim-pack XR-ODS III (75 mm × 2.0 mm, 1.6 μm)	[148]
UPLC-UV-MS/MS (242 nm)	*G. rhodantha*	Aerial	China	Metabolic fingerprinting analysis (loganic acid, mangiferin, sweroside, gentiopicroside, swertiamarin)	Methanol extraction (UE, RT, 30 min).	Mobile phase: A: 0.1% formic acid, B: acetonitrile. 0–1.62 min: 93–90% A, 1.62–14.72 min 90–74% A, 14.72–22.0 min: 74–20% A. Flow rate: 0.35 min/mL. Temperature: 40 °C Column: Shim-pack XR-ODS III (150 mm × 2.0 mm, 2.2 μm)	[11]
UPLC-UV-MS/MS (242 nm)	*G. rigescens*	Tissue culture materials of leaves	/	Analysis and determination of loganic acid, sweroside, swertiamarin, isoorientin, isovitexin, gentiopicroside	80% methanol extraction (UE, 35 min)	Mobile phase: A: 0.1% formic acid in water, B: methanol. 0–0.31 min: 13% B, 0.31–7.00 min: 20% B, 7.01–13.00 min: 46% B, 13.01–16.50 min: 83% B, 16.51–17.50 min: 90% B. Flow rate: 0.35 mL/min. Temperature: 40 °C Column: Shim-pack XR-ODS III (75 mm × 2.0 mm, 1.6 μm)	[107]
UPLC-TUV (250 nm)	*G*. *lutea*	Roots	Serbia	Quantification of gentiopicroside	Methanol, 96%, 75%, 50% and 25% ethanol and water extraction (RT, 48 h), respectively	Mobile phase: A: TFA (0.1% *v*/*v* in water), B: acetonitrile–methanol mixture (85:15 *v*/*v*). 0–7 min: B: 5% to 95%. Flow rate: 0.3 mL/min. Temperature: RT MS: positive and negative ion mode. Column: ACQUITY UPLCTM BEH C18 (1.7 μm,100 mm × 2.1 mm)	[149]
UPLC-UV-ESI/MS (242 nm, 320 nm)	*G*. *rhodantha*, *G. farreri*, *G*. *scabra*, *G. rigescens*	Aerial Roots and rhizomes	China	Simultaneous determination of six index constituents (loganic acid, gentiopicroside, ferulic acid, swertiamarin, mangiferin, sweroside)	70% methanol extraction (UE, 30 min)	Mobile phase: A: 0.1% formic acid water, B: acetonitrile. 0–2.5 min: A: 12%, 2.5–7.3 min: A: 12–15%, 7.3–12 min: A: 15–32%, 12–16 min: 32–78% A, 16–20 min: A: 12%. Flow rate: 0.25 mL/min. Temperature: 40 °C MS: negative and positive ion modes. Column: Shim-Pack XR-ODS III (150 mm × 2.0 mm, 2.2 μm).	[49]
UPLC-ESI-MS/MS	*G. rigescens*	Root, stem, leaf and seed	China	Simultaneous determination and detection of the four neuritogenic compounds (gentisides A, B, J and K)	95% ethanol extraction (UE, 40 min)	Mobile phase: methanol-0.1% formic acid in water = 95: 5 (*v*/*v*). Flow rate: 0.45 min/mL Temperature: 40 °C Column: Shim-pack XR–ODS III (75 mm × 2.0 mm, 1.6 μm)	[88]
UPLC-DAD/ESI-MS/MS (260, 320 nm)	*G. cruciata*	Aerial, Roots	East Serbia	Identified swertiamarin gentiopicrin, sweroside	Methanol extraction (soak, 24 h, three times)	Mobile phase: A: 0.1% formic acid in water. B: acetonitrile. 0–6 min: B: 5–20%, 6–10 min: B: 20–40%, 10–15 min: B: 40–50%, 15–17 min: B: 50–60%, 17–21 min: B: 60–95%, 21–23 min: B: 95%, 23–24 min: B: 95–5%, 24–30 min: B: 5%. Flow rate: 0.4 min/mL. Temperature: 30 °C MS: negative and positive ion modes Column: Hypersil gold C18 (50 mm × 2.1 mm, 1.9 μm)	[102]
UFLC-UV-ESI-MS/MS (242 nm)	*G. rigescens*	Roots and rhizomes	China	Optimization of ultrasonic extraction and determination of loganic acid, swertiamarin, sweroside gentiopicroside	UE with different conditions (extraction time, ratio of liquid to raw material, and methanol concentration)	Mobile phase: A: acetonitrile, B: 0.1% formic acid aqueous solution. 0–2.5 min: 12% A, 2.5–7.3 min: 12–15% A. Flow rate: 0.25 mL/min.Temperature: 40 °C Column: Shim-Pack XR-ODS III (150 mm × 2.0 mm, 2.2 μm)	[126]
HPLC-ESI-MS/MS	*G*. *kurroo*	/	Dachigam	Analysis of methanol extract; 11 compounds were identified.	Methanol extraction (soxhlet)	Mobile phase: A: aqueous formic acid (0.1%), B: 0.02% methanol solution. 0–8 min: 12% to 25% B, 8–12 min: 25% B, 12–16 min: 25–40% B, 16–40 min: 40–50% B, 40–50 min: 50–100% B. Flow rate: 1 mL/min. Column: Chomolith RP-18e column (4.6 mm × 50 mm, 5 μm) MS: positive and negative ion mode	[150]
LC-QTOF-MS/MS	*G. straminea*, *G*. *robusta*, *G. waltonii*, *G. lhassica*, *G. tibetica*	Roots	China	Chemical profiling of iridoids and secoiridoids	Ethanol extraction (UE, 30 min)	Mobile phase: A: acetonitrile, B: 0.02% formic acid aqueous solution. 0–4 min: 12% to 18% B, 4–12 min: 18% to 45% B, 12–20 min: 45% to 90% B. Flow rate: 0.3 mL/min. Temperature: 30 °C Column: Shiseido Spolar-C18 column (4.6 mm × 150 mm, 5 μm) MS: negative ion mode	[151]

DTGS: Deuterated triglycine sulfate detector; SGCC: silica gel column chromatography; PFG: pulsed-field gradient; BBO: multinuclear broadband observation; RT: room temperature; SDS: sodium dodecylsulfat.

**Table 5:** Applications of chemometric in *Gentiana* species.

Species	Method of Analysis	Chemometric Methods	Applications	Refs.
*Vitro* cultures of *G. triflora*	CE-MS	PCA, HCA, heat maps	a	[153]
*G. scabra*	IR	ICA	b	[89]
*G. scabra*	IR	MPLSR, SMLR	b	[67]
*G*. *rigescens*	IR	PCA-MD, PLS-DA	b	[152]
*G. macrophyllae*, *G. crassicaulis*, *G. straminea*, *G. dahurica*	IR	Cluster analysis	b	[139]
9 different species	FT-IR and UPLC-MS/MS	PCA	c	[50]
Tissue culture of *G. rigescens*	FT-IR and UPLC-MS/MS	PCA	a	[107]
*G. crassicaulis*, *G. macrophylla*	HPLC	PCA	b	[56]
*G. straminea*	HPLC	HCA	b	[88]
*G. lutea*	HPLC	PCA	b	[11]
*G. rigescens*, *G*. *scabra*	HPLC-DAD	SA, PCA	b	[46]
*G. rigescens*	UPLC-UV-MS/MS	SA, HCA heat maps PLS-DA	b	[149]
*G. rhodantha*	UPLC-UV-MS/MS	SA, PCA, PLS-DA	b	[17]
12 different species	HPLC-UV and HPLC-DAD-MS	SA, PCA	b	[23]
*G. rhodantha*, *G*. *farreri*, *G. scabra*, *G. rigescens*,	UPLC-UV-MS	SA, PCA	b	[49]
*G. macrophylla*, *G. straminea*, *G. crassicaulis*, *G. dahurica*, *G. officinalis*, *G. siphonantha*	HPLC	SA, HCA	d	[45]

Step-MLR or SMLR: Stepwise multiple linear regression, MPLSR: Modified partial least squares regression. Purpose of study: (a) metabolite analysis; (b) quality assessment; (c) taxonomic discrimination; (d) resource development.

## Supplementary Materials

Supplementary materials are available online.

## References

[1] Yang J.L., Liu L.L., Shi Y.P. (2010). Phytochemicals and biological activities of *Gentiana* species. Nat. Prod. Commun..

[2] Kim J.A., Son N.S., Son J.K., Jahng Y., Chang H.W., Jang T.S., Na M.K., Lee S.H. (2009). Two new secoiridoid glycosides from the rhizomes of *Gentiana scabra* Bunge. Arch. Pharm. Res..

[3] Li W., Kim J.H., Zhou W., Shim S.H., Ma J.Y., Kim Y.H. (2015). Soluble epoxide hydrolase inhibitory activity of phenolic components from the rhizomes and roots of *Gentiana* scabra. Biosci. Biotechnol. Biochem..

[4] Senol F.S., Orhan I.E. (2012). An in vitro perspective to cholinesterase inhibitory and antioxidant activity of five Gentiana species and Gentianella caucasea. Int. J. Food Sci. Nutr..

[5] Wang C., Wang Z., Wang W., Peng X. (2009). Advances in chemical components and pharmacology of genus Gentiana. Zhongguo Zhong Yao Za Zhi.

[6] Chinese Pharmacopeia Commission (2015). Pharmacopoeia of the People’s Republic of China, English Edition.

[7] Japanese Pharmacopeia Commission (2001). The Japanese Pharmacopoeia, Fourteenth Edition.

[8] Behera M.C., Raina R. (2011). Cytomorphology of Gentiana kurroo: An important endangered bitter plant of temperate Himalaya. J. For. Res..

[9] Arberas I., Leiton M.J., Domínguez J.B., Bueno J.M., Ariño A., Diego E.D., Renobales G., Renobales M.D. (1995). The volatile flavor of fresh *Gentiana lutea* L. Roots. Dev. Food Sci..

[10] Azman N.A.M., Gordon M.H., Skowyra M., Segovia F., Almajano M.P. (2014). Use of lyophilised and powdered *Gentiana lutea* root in fresh beef patties stored under different atmospheres. J. Sci. Food Agric..

[11] Mustafa A.M., Caprioli G., Ricciutelli M., Maggi F., Marín R., Vittori S., Sagratini G. (2015). Comparative HPLC/ESI-MS and HPLC/DAD study of different populations of cultivated, wild and commercial *Gentiana lutea* L.. Food Chem..

[12] Mustafa A.M., Maggi F., Öztürk N., Öztürk Y., Sagratini G., Torregiani E., Vittori S., Caprioli G. (2016). Chemical and biological analysis of the by-product obtained by processing *Gentiana lutea* L. and other herbs during production of bitter liqueurs. Ind. Crops Prod..

[13] Zając A., Pindel A. (2011). Review of the Willow Gentian, *Gentiana asclepiadea* L.. Biodiversity.

[14] Kesavan R., Chandel S., Upadhyay S., Bendre R., Ganugula R., Potunuru U.R., Giri H., Sahu G., Kumar U.P., Reddy B. (2016). *Gentiana lutea* exerts anti-atherosclerotic effects by preventing endothelial inflammation and smooth muscle cell migration. Nutr. Metab. Cardiovasc. Dis..

[15] Kušar A., Šircelj H., Baričevič D. (2013). Determination of seco-iridoid and 4-pyrone compounds in hydro-alcoholic extracts of *Gentiana lutea* L. subsp. symphyandra Murb. Leaves and roots by using high performance liquid chromatography. Isr. J. Plant Sci..

[16] Menković N., Savikinfodulović K., Savin K. (2000). Chemical composition and seasonal variations in the amount of secondary compounds in *Gentiana lutea* leaves and flowers. Planta Med..

[17] Pan Y., Zhang J., Shen T., Zhao Y.L., Wang Y.Z., Li W.Y. (2015). Comparative metabolic fingerprinting of Gentiana rhodantha from different geographical origins using LC-UV-MS/MS and multivariate statistical analysis. BMC Biochem..

[18] Kumar V., Chand R., Auzi A., Ikeshiro Y., Sarker S.D. (2003). 2′-(2,3-Dihydroxybenzoyloxy)-7-ketologanin: A novel iridoid glucoside from the leaves of Gentiana kurroo. Pharmazie.

[19] Wu Q.X., Liu X., Shi Y.P. (1979). Chemical components from Gentiana aristata. Chem. Biodivers..

[20] Xu M., Wang D. (2008). Iridoidal glucosides from Gentiana rhodantha. J. Asian Nat. Prod. Res..

[21] Sezik E., Aslan M., Yesilada E., Ito S. (2005). Hypoglycaemic activity of Gentiana olivieri and isolation of the active constituent through bioassay-directed fractionation techniques. Life Sci..

[22] Singh S., Yadav C., Noolvi M.N. (2012). Immunomodulatory activity of butanol fraction of Gentiana olivieri Griseb. On Balb/C mice. Asian Pac. J. Trop. Biomed..

[23] Liu F.-F., Wang Y.-M., Zhu H.-T., Dong W., Yang C.-R., Min X., Zhang Y.-J. (2014). Comparative Study on “Long-Dan”, “Qin-Jiao” and Their Adulterants by HPLC Analysis. Nat. Prod. Bioprospect..

[24] Aslan M., Orhan D.D., Orhan N. (2011). Effect of Gentiana olivieri on experimental epilepsy models. Pharmacogn. Mag..

[25] Glatz Z., Pospísilová J., Musil P. (2000). Determination of Gentiopicroside in extracts of centaurium erythreae and *Gentiana lutea* by micellar electrokinetic capillary chromatography. J. Liq. Chromatogr. Relat. Technol..

[26] Mansoor A., Samad A., Zaidi M.I., Aftab K. (1998). Hypotensive Effect of Gentiana olivieri and Its Alkaloid Gentianine in Rats. Pharm. Pharmacol. Commun..

[27] Mubashir K., Ghazanfar K., Ganai B.A., Akbar S., Malik A.H., Masood A. (2014). Scientific Validation of Gentiana kurroo Royle for Anti-Inflammatory and Immunomodulatory Potential. ISRN Inflamm..

[28] Qureshi R.A., Ghufran M.A., Gilani S.A., Sultana K., Ashraf M. (2007). Ethnobotanical studies of selected medicinal plants of Sudhan Gali and Ganga Chotti Hills, District Bagh, Azad Kashmir. Pak. J. Bot..

[29] Mihailović V., Vuković N., Nićiforović N., Solujić S., Mladenović M., Mašković P., Stanković M.S. (2011). Studies on the antimicrobial activity and chemical composition of the essential oils and alcoholic extracts of *Gentiana asclepiadea* L.. J. Med. Plant Res..

[30] Olennikov D.N., Kashchenko N.I., Chirikova N.K., Tankhaeva L.M. (2015). Iridoids and Flavonoids of Four Siberian Gentians: Chemical Profile and Gastric Stimulatory Effect. Molecules.

[31] Wang Y.M., Xu M., Wang D., Yang C.R., Zeng Y., Zhang Y.J. (2013). Anti-inflammatory compounds of “Qin-Jiao”, the roots of Gentiana dahurica (Gentianaceae). J. Ethnopharmacol..

[32] Wang Y.M., Xu M., Wang D., Zhu H.T., Yang C.R., Zhang Y.J. (2012). Review on “Long-Dan”, one of the traditional Chinese medicinal herbs recorded in Chinese pharmacopoeia. Nat. Prod. Bioprospect..

[33] Maurya A., Khan F., Bawankule D.U., Yadav D.K., Srivastava S.K. (2012). QSAR, docking and in vivo studies for immunomodulatory activity of isolated triterpenoids from Eucalyptus tereticornis and Gentiana kurroo. Eur. J. Pharm. Sci..

[34] Matsukawa K., Ogata M., Hikage T., Minami H., Shimotai Y., Saitoh Y., Yamashita T., Ouchi A., Tsutsumi R., Fujioka T. (2006). Antiproliferative activity of root extract from gentian plant (Gentiana triflora) on cultured and implanted tumor cells. Biosci. Biotechnol. Biochem..

[35] Mayorova O.Y., Hrytsak L.R., Drobyk N.M. (2015). The strategy of *Gentiana lutea* L. populations in the Ukrainian Carpathians. Russ. J. Ecol..

[36] Fiuk A., Rybczyński J.J. (2008). Factors influencing efficiency of somatic embryogenesis of Gentiana kurroo (Royle) cell suspension. Plant Biotechnol. Rep..

[37] Radanović D., Marković T., Aiello N., Fusani P. (2014). Cultivation trials on *Gentiana lutea* L. in Southern and South-eastern Europe. J. Appl. Res. Med. Aromat. Plants.

[38] Hudecová A., Hašplová K., Miadoková E., Magdolenová Z., Rinna A., Collins A.R., Gálová E., Vaculčíková D., Gregáň F., Dušinská M. (2012). *Gentiana asclepiadea* protects human cells against oxidation DNA lesions. Cell Biochem. Funct..

[39] Pan Y., Zhao Y.-L., Zhang J., Li W.-Y., Wang Y.-Z. (2016). Phytochemistry and Pharmacological Activities of the Genus Gentiana (Gentianaceae). Chem. Biodivers..

[40] Jia N., Li Y., Wu Y., Xi M., Hur G., Zhang X., Cui J., Sun W., Wen A. (2012). Comparison of the anti-inflammatory and analgesic effects of *Gentiana macrophylla* Pall. and Gentiana straminea Maxim., and identification of their active constituents. J. Ethnopharmacol..

[41] Mu Z., Yu Y., Gao H., Jiao W., Yao X. (2009). Chemical and pharmacological research for Sect. Aptera (gentiana). Zhongguo Zhong Yao Za Zhi.

[42] Liu C.X., Chen S.L., Xiao X.H., Zhang T.J., Hou W.B., Liao M.L. (2016). A new Concept on Quality Marker of Chinese Materia Medica: Quality Control for Chinese Medicinal Products. Chin. Tradit. Herb. Drugs.

[43] Yang W., Zhang Y., Wu W., Huang L., Guo D., Liu C. (2017). Approaches to establish Q-markers for the quality standards of traditional Chinese medicines. Acta Pharm. Sin. B.

[44] Zhou D., Hou Q., Si Q., Liu J., Yang H. (2010). Concentrations of the Active Constituents of the Tibetan Folk Medicine Qinjiao (Gentiana sect. Cruciata) within and between Taxonomic Species across the Qinghai-Tibetan Plateau. Chem. Biodivers..

[45] Cao X.Y., Wang Z.Z. (2010). Simultaneous determination of four iridoid and secoiridoid glycosides and comparative analysis of Radix Gentianae Macrophyllae and their related substitutes by HPLC. Phytochem. Anal..

[46] Duan B., Hu J., Huang L., Yang X., Chen F. (2012). Chemical fingerprint analysis of Gentianae Radix et Rhizoma by high-performance liquid chromatography. Acta Pharm. Sin. B.

[47] Wei S., Zhang P., Feng X., Kodama H., Yu C., Chen G. (2011). Qualitative and quantitative determination of ten iridoids and secoiridoids in Gentiana straminea Maxim. by LC-UV-ESI-MS. J. Nat. Med..

[48] Aberham A., Schwaiger S., Stuppner H., Ganzera M. (2007). Quantitative analysis of iridoids, secoiridoids, xanthones and xanthone glycosides in *Gentiana lutea* L. roots by RP-HPLC and LC-MS. J. Pharm. Biomed. Anal..

[49] Pan Y., Shen T., Zhang J., Zhao Y.L., Wang Y.Z., Li W.Y. (2015). Simultaneous determination of six index constituents and comparative analysis of four ethnomedicines from genus Gentiana using a UPLC-UV-MS method. Biomed. Chromatogr..

[50] Pan Y., Zhang J., Zhao Y.L., Wang Y.Z., Jin H. (2016). Chemotaxonomic Studies of Nine Gentianaceae Species from Western China Based on Liquid Chromatography Tandem Mass Spectrometry and Fourier Transform Infrared Spectroscopy. Phytochem. Anal..

[51] Carnat A., Fraisse D., Carnat A.P., Felgines C., Chaud D., Lamaison J.L. (2005). Influence of drying mode on iridoid bitter constituent levels in gentian root. J. Sci. Food Agric..

[52] Qi S.U., Shang P.P., Zhang Y.M., Jia N., Jiao H.E., Zhao W.N., Sun W.J. (2012). HPLC Fingerprint and LC-TOF-MS Analysis on Extract from Roots of *Gentiana macrophylla*. Chin. Herb. Med..

[53] Liang J., Ito Y., Zhang X., He J., Sun W. (2013). Rapid preparative separation of six bioactive compounds from Gentiana crassicaulis Duthie ex Burk. using microwave-assisted extraction coupled with high-speed counter-current chromatography. J. Sep. Sci..

[54] Aberham A., Pieri V., Croom E.M., Ellmerer E., Stuppner H. (2011). Analysis of iridoids, secoiridoids and xanthones in *Centaurium erythraea*, Frasera caroliniensis and *Gentiana lutea* using LC-MS and RP-HPLC. J. Pharm. Biomed. Anal..

[55] Hayta S., Akgun I.H., Ganzera M., Bedir E., Gurel A. (2011). Shoot proliferation and HPLC-determination of iridoid glycosides in clones of *Gentiana cruciata* L.. Plant Cell Tissue Organ Cult..

[56] Wang Y., Ahmad B., Duan B., Rui Z., Huang L. (2016). Chemical and genetic comparative analysis of Gentiana crassicaulis and *Gentiana macrophylla*. Chem. Biodivers..

[57] Suh H.W., Lee K.B., Kim K.S., Yang H.J., Choi E.K., Min H.S., Yong S.P., Na Y.C., Ahn K.S., Jang Y.P. (2015). A bitter herbal medicine *Gentiana scabra* root extract stimulates glucagon-like peptide-1 secretion and regulates blood glucose in db/db mouse. J. Ethnopharmacol..

[58] Hayta S., Gurel A., Akgun I.H., Altan F., Ganzera M., Tanyolac B., Bedir E. (2011). Induction of *Gentiana cruciata* hairy roots and their secondary metabolites. Biologia.

[59] Jia N., Wei C., Li Y., Ding L., Duan J., Jia C., Cao S., Zhao C., Wu Y., Wen A. (2016). Iridoid glycosides from the flowers of *Gentiana macrophylla* Pall. ameliorate collagen-induced arthritis in rats. J. Ethnopharmacol..

[60] Wu W., Ye H., Tang M., Peng A., Shi J., Li S., Zhong S., He S., Lai H., Zhao J. (2012). Using High-Performance Counter-Current Chromatography Combined with Preparative High Performance Liquid Chromatogramphy for the Separation of Bioactive Compounds from the Water Extract of *Gentiana macrophylla* Pall. Sep. Sci. Technol..

[61] Chen Y., Qiu D.Y., Guo F.X., Wang E.J., Liu F.Z. (2007). Investigation on explovitage of Gentiana straminea. J. Chin. Med. Mater..

[62] Liang X., Tian Q., Wei Z., Liu F.E., Chen J., Zhao Y., Qu P., Huang X., Zhou X., Liu N. (2011). Effect of Feining on bleomycin-induced pulmonary injuries in rats. J. Ethnopharmacol..

[63] Huang S.H., Chen C.F., Wu C.T., Kuo C.L., Tsay H.S. (2013). Comparative analysis among three Taiwan-specific Gentiana species and Chinese medicinal plant *Gentiana scabra*. Bot. Stud..

[64] Keller F. (1986). Gentiopicroside is Located in the Vacuoles of Root Protoplasts of *Gentiana lutea*. J. Plant Physiol..

[65] Branka V., Dijana K., Teodora J., Snežana Z., Dragan V. (2013). Quantitative determination of secoiridoid and xanthone glycosides of Gentiana dinarica Beck cultured in vitro. Acta Physiol. Plant..

[66] Jiang R.W., Wong K.L., Chan Y.M., Xu H.X., But P.H., Shaw P.C. (2005). Isolation of iridoid and secoiridoid glycosides and comparative study on Radix gentianae and related adulterants by HPLC analysis. Phytochemistry.

[67] Chuang Y.K., Chen S., Lo Y.M., Yang I.C., Cheng Y.F., Wang C.Y., Tsai C.Y., Hsieh R.M., Wang K.H., Lai C.C. (2013). Quantification of bioactive gentiopicroside in the medicinal plant *Gentiana scabra* Bunge using near infrared spectroscopy. J. Food Drug Anal..

[68] Vanhaelen M., Vanhaelen-Fastre R. (1983). Quantitative determination of biologically active constituents in medicinal plant crude extracts by thin-layer chromatography—Densitometry: I. *Aesculus hippocastaneum* L., Arctostaphyllos uva-ursi Spreng, Fraxinus excelsior L., *Gentiana lutea* L., Gly. J. Chromatogr..

[69] Hayashi T., Matsuda T., Yoneda K. (1976). Studies on crude drugs originated from Gentianaceous plants. VI. Contents of gentiopicroside in various parts of *Gentiana scabra* and accumulation of gentiopicroside in Gentiana triflora. Yakugaku Zasshi J. Pharm. Sci. Jpn..

[70] Hayashi T., Higashino M. (1976). Studies on crude drugs originated from gentianaceous plants. III. The bitter principle of the Chinese crude drug qinjiao and its content (author's transl). Yakugaku zasshi: J. Pharm. Sci. Jpn..

[71] Hayashi T. (1976). Studies on crude drugs originated from Gentianaceous plants. I. Determination of gentiopicroside, the bitter principle of Gentianae radix and Gentianae scabrae radix (author’s transl). Yakugaku zasshi. J. Pharm. Sci. Jpn..

[72] Öztürk N., Ba K.H., Aydin S., Öztürk Y., Çali I. (2002). Effects of *Gentiana lutea* ssp. symphyandra on the Central Nervous System in Mice. Phytother. Res..

[73] Schaufelberger D., Hostettmann K. (1987). High-performance liquid chromatographic analysis of secoiridoid flavone glycosides in closely related Gentiana species. J. Chromatogr. A.

[74] Liu H., Wang K., Zhao Y., Zhang H., Chen X., Hu Z. (2000). Identification and Determination of Active Components in Gentiana rigescens Franch by Micellar Electrokinetic Chromatography. J. High. Resolut. Chromatogr..

[75] Zhao S., Liu Q., Chen X., Hu Z. (2004). Separation and determination of gentiopicroside and swertiamarin in Tibetan medicines by micellar electrokinetic electrophoresis. Biomed. Chromatogr..

[76] Szucs Z., Dános B., Nyiredy S. (2002). Comparative analysis of the underground parts of Gentiana species by HPLC with diode-array and mass spectrometric detection. Chromatographia.

[77] Fan H., Zang Y., Zhang Y., Zhang H.F., Zhao Z., Hu J.F. (2010). Triterpenoids and Iridoid Glycosides from Gentiana dahurica. Helv. Chim. Acta.

[78] Hudecová A., Kusznierewicz B., Hašplová K., Huk A., Magdolenová Z., Miadoková E., Gálová E., Dušinská M. (2012). *Gentiana asclepiadea* exerts antioxidant activity and enhances DNA repair of hydrogen peroxide- and silver nanoparticles-induced DNA damage. Food Chem. Toxicol..

[79] Quercia V., Battaglino G., Pierini N., Turchetto L. (1980). Determination of the bitter constituents of the Gentiana root by high-performance liquid chromatography. J. Chromatogr. A.

[80] Mihailović V., Mihailović M., Uskoković A., Arambašić J., Mišić D., Stanković V., Katanić J., Mladenović M., Solujić S., Matić S. (2013). Hepatoprotective effects of *Gentiana asclepiadea* L. extracts against carbon tetrachloride induced liver injury in rats. Food Chem. Toxicol..

[81] Mihailović V., Katanić J., Mišić D., Stanković V., Mihailović M., Uskoković A., Arambašić J., Solujić S., Mladenović M., Stanković N. (2014). Hepatoprotective effects of secoiridoid-rich extracts from *Gentiana cruciata* L. against carbon tetrachloride induced liver damage in rats. Food Funct..

[82] Orhan D.D., Aslan M., Aktay G., Ergun E., Yesilada E., Ergun F. (2003). Evaluation of hepatoprotective effect of Gentiana olivieri herbs on subacute administration and isolation of active principle. Life Sci..

[83] Toker G., Edis M., Yeşilada E. (2011). Quantitative analysis of isoorientin in several Turkish Gentiana species by high performance liquid chromatography. Fabad J. Pharm. Sci..

[84] Wu Q.X., Chen J., Shi Y.P. (2010). RP-HPLC and NMR study of antioxidant flavonoids in extract from Gentiana piasezkii. J. Anal. Chem..

[85] Hudecová A., Kusznierewicz B., Rundénpran E., Magdolenová Z., Hasplová K., Rinna A., Fjellsbø L.M., Kruszewski M., Lankoff A., Sandberg W.J. (2012). Silver nanoparticles induce premutagenic DNA oxidation that can be prevented by phytochemicals from *Gentiana asclepiadea*. Mutagenesis.

[86] Liu B., Yue M.E., Yang S.R., Shi Y.P. (2005). Determination of Phenolic Glucosides in Gentiana piasezkii by Capillary Zone Electrophoresis. Chromatographia.

[87] Singh S., Yadav C.P., Noolvi M.N. (2012). Quantification of Oleanolic acid in the flower of Gentiana olivieri Griseb. by HPLC. J. Basic Clin. Pharm..

[88] Yang H., Que S., Huang H., Liu H., Jiang Y. (2014). The Chemical Components of Volatile Oils in Gentiana nubigenan Edgew. Med. Plant.

[89] Chuang Y.K., Yang I.C., Lo Y.M., Tsai C.Y., Chen S. (2014). Integration of independent component analysis with near-infrared spectroscopy for analysis of bioactive components in the medicinal plant *Gentiana scabra* Bunge. J. Food Drug Anal..

[90] Citová I., Ganzera M., Stuppner H., Solich P. (2008). Determination of gentisin, isogentisin, and amarogentin in *Gentiana lutea* L. by capillary electrophoresis. J. Sep. Sci..

[91] Pan Y., Shen T., Pan J., Xiao D., Li Z., Li W., Wang Y. (2014). Development and validation of a UPLC-MS/MS method for the simultaneous determination and detection of four neuritogenic compounds in different parts of Franch using multiple reaction monitoring and precursor ion scanning. Anal. Methods.

[92] Cai W., Xu H., Xie L., Sun J., Sun T., Wu X., Fu Q. (2016). Purification, characterization and in vitro anticoagulant activity of polysaccharides from *Gentiana scabra* Bunge roots. Carbohydr. Polym..

[93] Wang Z., Wang C., Su T., Zhang J. (2014). Antioxidant and immunological activities of polysaccharides from *Gentiana scabra* Bunge roots. Carbohydr. Polym..

[94] Cheng Z., Zhang Y., Song H., Zhou H., Zhong F., Hu H., Feng Y. (2016). Extraction optimization, characterization and antioxidant activity of polysaccharide from *Gentiana scabra* bge. Int. J. Biol. Macromol..

[95] Li X., Zhou J.Y., Yang Q.W., Chen Y., Piao Y.A., Li H.Y. (2011). Inhibition activities of polysaccharide (RG4-1) from Gentiana rigescens against RSV. J. Asian Nat. Prod. Res..

[96] Zhang J., Yuan T., Wang Y., Zhao Y., Zhang J., Jin H. (2012). Determination of Mineral Elements in Gentiana rigescens from Different Zones of Yunnan, China. Biol. Trace Elem. Res..

[97] Radanovic D., Anticmladenovic S., Jakovljevic M., Kresovic M. (2007). Content of heavymetals in *Gentiana lutea* L. roots and galenic forms. J. Serb. Chem. Soc..

[98] Zeiner M., Cindrić I.J., Požgaj M., Pirkl R., Šilić T., Stingeder G. (2014). Influence of soil composition on the major, minor and trace metal content of Velebit biomedical plants. J. Pharm. Biomed. Anal..

[99] Niu X.X., Chen X.W., Su H., Eneji A.E., Guo Y.H., Dong X.H. (2014). Changes of Secondary Metabolites and Trace Elements in *Gentiana macrophylla* Flowers: A Potential Medicinal Plant Part. Chin. Herb. Med..

[100] Sun J., Chen G., Zhao X., Xu W., Zhou G., Han Y., You J. (2007). Determination of 30 Free Fatty Acids in Two Famous Tibetan Medicines by HPLC with Fluorescence Detection and Mass Spectrometric Identification. Chromatographia.

[101] Sun J., Li F., Xu W., Zhou G., You J., Chen G. (2009). LC-ESI-MS Determination of 20 Free Amino Acids in Tibetan Medicine Gentiana dahurica with Pre-Column Fluorescence Derivatization. Chromatographia.

[102] Mihailović V., Mišić D., Matić S., Mihailović M., Stanić S., Vrvić M.M., Katanić J., Mladenović M., Stanković N., Boroja T. (2015). Comparative phytochemical analysis of *Gentiana cruciata* L. roots and aerial parts, and their biological activities. Ind. Crops Prod..

[103] Mihailovic V., Matic S., Mišic D., Solujic S., Stanic S., Katanic J., Mladenovic M., Stankovic N. (2013). Chemical composition, antioxidant and antigenotoxic activities of different fractions of *Gentiana asclepiadea* L. roots extract. Excli J..

[104] Bao Y.F., Ji-Yu L.I., Zheng L.F., Hong-Yu L.I. (2015). Antioxidant activities of cold-nature Tibetan herbs are signifcantly greater than hot-nature ones and are associated with their levels of total phenolic components. Chin. J. Nat. Med..

[105] Wani B.A., Ramamoorthy D., Rather M.A., Arumugam N., Qazi A.K., Majeed R., Hamid A., Ganie S.A., Ganai B.A., Anand R. (2013). Induction of apoptosis in human pancreatic MiaPaCa-2 cells through the loss of mitochondrial membrane potential (Δ Ψ m) by Gentiana kurroo root extract and LC-ESI-MS analysis of its principal constituents. Phytomedicine.

[106] Rossetti V., Lombard A., Buffa M., Sancin P., Borgarello E. (2008). Seasonal Variations in Components of Dried Roots from the Western Alpine Region. Pharm. Biol..

[107] Pan Y., Zhang J., Zhao Y.L., Wang Y.Z., Huang H.Y. (2015). Investigation of metabolites accumulation in medical plant Gentiana rigescens during different growing stage using LC-MS/MS and FT-IR. Bot. Stud..

[108] Georgieva E., Handjieva N., Popov S., Evstatieva L. (2005). Comparative analysis of the volatiles from flowers and leaves of three Gentiana species. Biochem. Syst. Ecol..

[109] Chialva F., Frattini C., Martelli A. (1985). Unusual essential oils with aromatic properties III. Volatile components of gentian roots. Z. Lebensm. Unters. Forsch.

[110] Jaemin L., Sugawara E., Yokoi S., Takahata Y. (2008). Genotypic variation of volatile compounds from flowers of gentians. Breed. Sci..

[111] Mustafa A.M., Caprioli G., Maggi F., Vittori S., Sagratini G. (2016). Comparative Analysis of the Volatile Profiles from Wild, Cultivated, and Commercial Roots of *Gentiana lutea* L. by Headspace Solid Phase Microextraction (HS-SPME) Coupled to Gas Chromatography Mass Spectrometry (GC-MS). Food Anal. Methods.

[112] Wani B.A., Ramamoorthy D., Rather M.A., Ganai B.A., Masood A., Zargar M.A., Wani I. (2011). Headspace solid-phase microextraction (HS-SPME) Gas Chromatography Mass Spectrometric (GC-MS) analysis of the volatile aroma components of Gentiana kurroo Royle. J. Pharm..

[113] Qi L.W., Wang C.Z., Yuan C.S. (2011). Isolation and analysis of ginseng: advances and challenges. Nat. Prod. Rep..

[114] Guo Y., Lu Y. (1983). Studies on the transformation of gentiopicroside to gentianal. Chin. J. Pharm. Anal..

[115] Yin H., Zhao Q., Sun F.M., An T. (2009). Gentiopicrin-producing endophytic fungus isolated from *Gentiana macrophylla*. Phytomedicine.

[116] Ariño A., Arberas I., Leiton M.J., Renobales M.D., Dominguez J.B. (1997). The extraction of yellow gentian root (*Gentiana lutea* L.). Z. Lebensm. Unters. Forsch.

[117] Hao C.Q., Guo H.Y., Leng X.H., Jun L.I., Chen H.Y. (2013). Optimization of the Dynamic Reflux Extraction Technology of Gentiopicroside from the Roots of *Gentiana macrophylla* by Orthogonal Experiment. J. Northwest For. Univ..

[118] Nickerson G.B., Likens S.T. (1966). Gas Chromatography evidence for the occurrence of hop oil components in beer. J. Chromatogr. A.

[119] Yang H., Liu J., Chen S., Hu F., Zhou D. (2014). Spatial variation profiling of four phytochemical constituents in Gentiana straminea (Gentianaceae). J. Nat. Med..

[120] Teixeira S., Mendes A., Alves A., Santos L. (2007). Simultaneous distillation-extraction of high-value volatile compounds from *Cistus ladanifer* L.. Anal. Chim. Acta.

[121] Victório C.P., Riehl C.A.D.S., Lage C.L.S. (2009). Simultaneous Distillation-Extraction, Hydrodistillation and Static Headspace Methods for the Analysis of Volatile Secondary Metabolites of Alpinia zerumbet (Pers.) Burtt et Smith. from Southeast Brazil. J. Essent. Oil Bear. Plants.

[122] Sides A., Robards K., Helliwell S. (2000). Developments in extraction techniques and their application to analysis of volatiles in foods. TrAC Trends Anal. Chem..

[123] Watkins P.J., Rose G., Warner R.D., Dunshea F.R., Pethick D.W. (2012). A comparison of solid-phase microextraction (SPME) with simultaneous distillation-extraction (SDE) for the analysis of volatile compounds in heated beef and sheep fats. Meat Sci..

[124] Hao C.Q., Guo H.Y., Chen H.Y., Liu M.J. (2012). Effects of Ethanol Concentration on the Extraction Rate of Gentiopicroside from the *Gentiana macrophylla* Pall in Ningxia Liupan Mountain of China. Med. Plants.

[125] Savikin K., Menkovic N., Zdunic G., Stevic T., Radanovic D., Jankovic T. (2009). Antimicrobial activity of *Gentiana lutea* L. extracts. Z. Naturforsch. C Biosci..

[126] Pan Y., Zhang J., Shen T., Zuo Z.T., Jin H., Wang Y.Z., Li W.Y. (2015). Optimization of ultrasonic extraction by response surface methodology combined with ultrafast liquid chromatography–ultraviolet method for determination of four iridoids in Gentiana rigescens. J. Food Drug Anal..

[127] Yang L., Wang H., He T. (2014). Determination of gentiopicroside contents during somatic embryogenesis in Gentiana straminea Maxim. Acta Physiol. Plant..

[128] Wang C., Wang Y., Zhang J., Wang Z. (2014). Optimization for the extraction of polysaccharides from *Gentiana scabra* Bunge and their antioxidant in vitro and anti-tumor activity in vivo. Taiwan Inst. Chem. Eng..

[129] Bettinelli M., Beone G.M., Spezia S., Baffi C. (2000). Determination of heavy metals in soils and sediments by microwave-assisted digestion and inductively coupled plasma optical emission spectrometry analysis. Anal. Chim. Acta.

[130] Umagat H., Wen K.F. (1982). Total amino acid analysis using pre-column fluorescence derivatization. J. Chromatogr. A.

[131] Chen G., Wei S.H., Yu C.Y. (2009). Secoiridoids from the roots of Gentiana straminea. Biochem. Syst. Ecol..

[132] Hostettmann K. (1980). Droplet counter-current chromatography and its application to the preparative scale separation of natural products. Planta Med..

[133] Hostettmann K., Hostettmann-Kaldas M., Sticher O. (1979). Application of droplet counter-current chromatography to the isolation of natural products. J. Chromatogr. A.

[134] Rho T., Jung M., Lee M.W., Chin Y.W., Yoon K.D. (2016). Efficient methods for isolating five phytochemicals from *Gentiana macrophylla* using high-performance countercurrent chromatography. J. Sep. Sci..

[135] Liang Y., Hu J., Chen H., Zhang T., Ito Y. (2007). Preparative Isolation and Purification of Four Compounds from Chinese Medicinal Herb *Gentiana scabra* Bunge by High-Speed Countercurrent Chromatography. J. Liq. Chromatogr. Relat. Technol..

[136] Baek S.H., Bae O.N., Park J.H. (2012). Recent methodology in ginseng analysis. J. Ginseng Res..

[137] Wang Y., Choi H.K., Brinckmann J.A., Xue J., Huang L. (2015). Chemical analysis of Panax quinquefolius (North American ginseng): A review. J. Chromatogr. A.

[138] Marston A., Hostettmann K. (2006). Developments in the application of counter-current chromatography to plant analysis. J. Chromatogr. A.

[139] Zeng R., Liu H., Qiong L.I., Zhang H., Yan Q.U. (2013). Infrared Fingerprint Analysis of Gentianae macrophyllae Radix Coupled with Sequential Analysis of Dual-indexes and Cluster Analysis. Med. Plant.

[140] Pan Y., Zhang J., Shen T., Zhao Y.L., Zuo Z.T., Wang Y.Z., Li W.Y. (2015). Liquid Chromatography Tandem Mass Spectrometry Combined with Fourier Transform Mid-Infrared Spectroscopy and Chemometrics for Comparative Analysis of Raw and Processed Gentiana rigescens. J. Liq. Chromatogr. Relat. Technol..

[141] Tanaka R., Hasebe Y., Nagatsu A. (2014). Application of quantitative ^1^H-NMR method to determination of gentiopicroside in Gentianae radix and Gentianae scabrae radix. J. Nat. Med..

[142] Li Z., Welbeck E., Yang L., He C., Hu H., Song M., Bi K., Wang Z. (2014). A quantitative 1 H nuclear magnetic resonance (qHNMR) method for assessing the purity of iridoids and secoiridoids. Fitoterapia.

[143] Takahashi H., Imamura T., Miyagi A., Uchimiya H. (2012). Comparative metabolomics of developmental alterations caused by mineral deficiency during in vitro culture of Gentiana triflora. Metabolomics.

[144] Koshioka M., Miyamoto K., Horio T., Namura S., Hisamatsu T., Kubota S., Ernstsen A., Junttila O., Mander L.N. (1998). Identification of endogenous gibberellins in stems and leaves in vegetative growth stage of Gentiana triflora. J. Plant Physiol..

[145] Zheng P., Zhang K., Wang Z. (2011). Genetic diversity and gentiopicroside content of four Gentiana species in China revealed by ISSR and HPLC methods. Biochem. Syst. Ecol..

[146] Sheu M.J., Chiu C.C., Yang D.J., Hsu T.C., Tzang B.S. (2017). The Root Extract of *Gentiana macrophylla* Pall. Alleviates B19-NS1-Exacerbated Liver Injuries in NZB/W F1 Mice. J. Med. Food.

[147] Wang S., Xu Y., Chen P., Zhang Y. (2014). Structural characterization of secoiridoid glycosides by high-performance liquid chromatography/electrospray ionization mass spectrometry. Rapid Commun. Mass Spectrom..

[148] Pan Y., Zhang J., Shen T., Zhao Y.L., Zuo Z.T., Wang Y.Z., Li W.Y. (2016). Investigation of chemical diversity in different parts and origins of ethnomedicine Gentiana rigescens Franch using targeted metabolite profiling and multivariate statistical analysis. Biomed. Chromatogr..

[149] Nastasijević B., Lazarević-Pašti T., Dimitrijević-Branković S., Pašti I., Vujačić A., Joksić G., Vasić V. (2012). Inhibition of myeloperoxidase and antioxidative activity of *Gentiana lutea* extracts. J. Pharm. Biomed. Anal..

[150] Mubashir K., Ganai B.A., Ghazanfar K., Akbar S., Rah B., Tantry M., Masood A. (2017). Anti-inflammatory and immuno-modulatory studies on LC-MS characterised methanol extract of Gentiana kurroo Royle. BMC Complement. Altern. Med..

[151] Wu J., Zhao Z., Wu L., Wang Z. (2016). Authentication of Gentiana straminea Maxim. and its substitutes based on chemical profiling of iridoids using liquid chromatography with mass spectrometry. Biomed. Chromatogr. BMC.

[152] Zhao Y., Zhang J., Jin H., Zhang J., Shen T., Wang Y. (2015). Discrimination of Gentiana rigescens from Different Origins by Fourier Transform Infrared Spectroscopy Combined with Chemometric Methods. J. AOAC Int..

[153] Rosen A.L., Hieftje G.M. (2004). Inductively coupled plasma mass spectrometry and electrospray mass spectrometry for speciation analysis: applications and instrumentation. Spectrochim. Acta Part B.

[154] Odontuya G., Hoult J., Houghton P. (2005). Structure-activity relationship for antiinflammatory effect of luteolin and its derived glycosides. Phytother. Res..

[155] Tranchida P.Q., Franchina F.A., Dugo P., Mondello L. (2016). Comprehensive two-dimensional gas chromatography-mass spectrometry: Recent evolution and current trends. Mass Spectrom. Rev..

[156] Marston A. (2007). Role of advances in chromatographic techniques in phytochemistry. Phytochemistry.

[157] Öztürk N., Herekman-Demir T., Öztürk Y., Bozan B., Başer K.H.C. (1998). Choleretic activity of *Gentiana lutea* ssp. symphyandra in rats. Phytomedicine.

[158] Csupor D., Csorba A., Hohmann J. (2016). Recent advances in the analysis of flavonolignans of Silybum marianum. J. Pharm. Biomed. Anal..

[159] Loos G., Van Schepdael A., Cabooter D. (2016). Quantitative mass spectrometry methods for pharmaceutical analysis. Philos. Trans. R. Soc. A.

[160] Wang C.S., Dong H.J., Bao Y.T., Chen X.H., Hai Y., Zeng R., Pharmacy S.O. (2016). Rapid analysis on chemical constituents from roots of Gentiana crasicaulis by ultra-high performance liquid chromatography coupled with hybrid quadrupole-orbitrap mass spectrometry. Chin. Tradit. Herb. Drugs.

[161] Gómezcaravaca A.M., Maggio R.M., Cerretani L. (2016). Chemometric applications to assess quality and critical parameters of virgin and extra-virgin olive oil. A review. Anal. Chim. Acta.

[162] Gad H.A., El-Ahmady S.H., Abou-Shoer M.I., Al-Azizi M.M. (2013). Application of chemometrics in authentication of herbal medicines: a review. Phytochem. Anal..

[163] Martín-Alberca C., Ortega-Ojeda F.E., García-Ruiz C. (2016). Analytical tools for the analysis of fire debris. A review: 2008–2015. Anal. Chim. Acta.

[164] Yi L., Dong N., Yun Y., Deng B., Ren D., Liu S., Liang Y. (2016). Chemometric methods in data processing of mass spectrometry-based metabolomics: A review. Anal. Chim. Acta.

