# Supplementary material for: Analytical Methods of Phytochemicals from the Genus Gentiana
Source: Molecules. 2017 Nov 28;22(12):2080. doi: 10.3390/molecules22122080 (PMC6149888; doi:10.3390/molecules22122080)
Supplement: Supplementary file 1 [file molecules-22-02080-s001.pdf]

Table S1. The information of plants which checked in Plant List databases

Name
Gentiana acaulis L.
Gentiana affinis Griseb.
Gentiana affinis subsp. rusbyi (Greene ex Kusn.) Halda
Gentiana alata T.N.Ho
Gentiana alba Muhl.
Gentiana albicalyx Burkill
Gentiana albicalyx subsp. globosa (T.N.Ho) Halda
Gentiana albomarginata C.Marquand
Gentiana albomarginata subsp. scytophylla (T.N.Ho) Halda
Gentiana algida Pall.
Gentiana algida var. purdomii (C.Marquand) T.N.Ho
Gentiana alii (Omer & Qaiser) T.N.Ho
Gentiana alpina Vill.
Gentiana alpina var. transiens Nègre
Gentiana alsinoides Franch.
Gentiana alsinoides subsp. scabrifilamenta (T.N.Ho) Halda
Gentiana altigena Harry Sm.
Gentiana altorum Harry Sm. ex C.Marquand
Gentiana × ambigua Hayek
Gentiana amplicrater Burkill
Gentiana andrewsii Griseb.
Gentiana andrewsii var. dakotica A.Nelson
Gentiana angustifolia Vill.
Gentiana anisostemon C.Marquand
Gentiana annaverae Pînzaru
Gentiana aperta Maxim.
Gentiana apiata N.E.Br.
Gentiana aquatica L.
Gentiana aquatica var. baltistanica (Omer) Halda
Gentiana aquatica var. bomiensis (T.N.Ho) Halda
Gentiana aquatica subsp. laeviuscula (Ohwi) Ohwi
Gentiana aquatica var. pseudoaquatica (Kusn.) S.Agrawal
Gentiana arenicola Kerr
Gentiana arethusae Burkill
Gentiana arethusae subsp. delicatula (C.Marquand) Halda
Gentiana argentea (Royle ex D.Don) Royle ex D.Don
Gentiana arisanensis Hayata
Gentiana aristata Maxim.
Gentiana aristata subsp. asparagoides (T.N.Ho) Halda
Gentiana asclepiadea L.
Gentiana asterocalyx Diels
Gentiana atlantica Litard. & Maire
Gentiana atuntsiensis W.W.Sm.

Gentiana austromontana J.S.Pringle & Sharp
Gentiana autumnalis L.
Gentiana autumnalis subsp. pennelliana (Fernald) Halda
Gentiana axilliflora Levl. & Vaniot
Gentiana baeuerlenii L.G.Adams
Gentiana baeuerlenii subsp. bredboensis (L.G.Adams) Halda
Gentiana bambuseti T.Y.Hsieh, T.C.Hsu, S.M.Ku & C.I Peng
Gentiana bavarica L.
Gentiana bavarica var. subacaulis (Wahlenb.) Schleich.
Gentiana beamanii J.S.Pringle
Gentiana bella Franch. ex Hemsl.
Gentiana bicuspidata (G.Don) Briq.
Gentiana × billingtonii Farw.
Gentiana boissieri Schott & Kotschy ex Boiss.
Gentiana bokorensis Hul
Gentiana borneensis Hook.f.
Gentiana borneensis subsp. apoensis (Merr.) Halda
Gentiana borneensis subsp. malayana (Ridl.) Halda
Gentiana boryi Boiss.
Gentiana brachyphylla Vill.
Gentiana brachyphylla subsp. favratii (Rittener) Tutin
Gentiana brentae Prosser & Bertolli
Gentiana bryoides Burkill
Gentiana burseri Lapeyr.
Gentiana burseri subsp. villarsii (Griseb.) Rouy
Gentiana cachemirica Decne.
Gentiana caelestis (C.Marquand) Harry Sm.
Gentiana caeruleogrisea T.N.Ho
Gentiana caliculata Lex.
Gentiana calycosa Griseb.
Gentiana capitata Buch.-Ham. ex D.Don
Gentiana capitata subsp. harwanensis (G.Singh) Halda
Gentiana carinata (D.Don) Griseb.
Gentiana carinicostata Wernham
Gentiana carinicostata subsp. sclerophylla (P.Royen) Halda
Gentiana caryophyllea Harry Sm.
Gentiana catesbaei Walter
Gentiana cephalantha Franch. ex Hemsl.
Gentiana cephalantha subsp. vaniotii (H.Le$Av.) Halda
Gentiana cephalodes Edgew.
Gentiana × charpentieri Thomas ex Hegetschw.
Gentiana chateri T.N.Ho
Gentiana chinensis Kusn.
Gentiana choanantha C.Marquand
Gentiana choanantha subsp. curvianthera (T.N.Ho) Halda
Gentiana chosenica Okuyama

Gentiana chungtienensis C.Marquand
Gentiana chungtienensis subsp. subuniflora (C.Marquand) Halda
Gentiana clarkei Kusn.
Gentiana clarkei subsp. conduplicata (T.N.Ho) Halda
Gentiana clausa Raf.
Gentiana clusii Perr. & Songeon
Gentiana clusii subsp. rochelii (A.Kern.) Halda
Gentiana confertifolia C.Marquand
Gentiana coronata (D.Don ex Royle) Griseb.
Gentiana crassa Kurz
Gentiana crassa subsp. rigescens (Franch. ex Hemsl.) Halda
Gentiana crassicaulis Duthie ex Burkill
Gentiana crassula Harry Sm.
Gentiana crassuloides Bureau & Franch.
Gentiana crassuloides subsp. curviphylla (T.N.Ho) Halda
Gentiana crassuloides subsp. mailingensis (T.N.Ho) Halda
Gentiana crassuloides subsp. nyingchiensis (T.N.Ho) Halda
Gentiana cristata Harry Sm.
Gentiana cruciata L.
Gentiana cruttwellii Harry Sm.
Gentiana cuneibarba Harry Sm.
Gentiana × curtisii J.S.Pringle
Gentiana dahurica Fisch.
Gentiana damyonensis C.Marquand
Gentiana damyonensis subsp. hicksii (Harry Sm.) Halda
Gentiana davidii Franch.
Gentiana davidii var. formosana (Hayata) T.N.Ho
Gentiana davidii var. fukienensis (Y.Ling) T.N.Ho
Gentiana decemfida Buch.-Ham. ex D.Don
Gentiana decora Pollard
Gentiana decorata Diels
Gentiana decorata subsp. leucantha (Harry Sm.) Halda
Gentiana decumbens L.f.
Gentiana delavayi Franch.
Gentiana deltoidea Harry Sm.
Gentiana dendrologi C.Marquand
Gentiana densiflora T.N.Ho
Gentiana depressa D.Don
Gentiana × digenea Jakow.
Gentiana dinarica Beck
Gentiana divaricata T.N.Ho
Gentiana diversifolia Merr.
Gentiana douglasiana Bong.
Gentiana doxiongshangensis T.N.Ho
Gentiana dschungarica Regel
Gentiana duclouxii Franch.

Gentiana durangensis Villarreal
Gentiana ecaudata C.Marquand
Gentiana elmeriana Halda
Gentiana elwesii C.B.Clarke
Gentiana emodi C.Marquand ex Sealy
Gentiana ettingshausenii F.Muell.
Gentiana exigua Harry Sm.
Gentiana expansa Harry Sm.
Gentiana faucipilosa Harry Sm.
Gentiana fieldiana J.S.Pringle
Gentiana filistyla Balf.f. & Forrest
Gentiana flavomaculata Hayata
Gentiana flavomaculata subsp. tatakensis (Masam.) Halda
Gentiana flexicaulis Harry Sm.
Gentiana flexicaulis var. complexa (T.N.Ho) Halda
Gentiana formosa Harry Sm.
Gentiana forrestii C.Marquand
Gentiana forrestii subsp. yakumontana (Masam.) Halda
Gentiana franchetiana Kusn.
Gentiana fremontii Torr.
Gentiana frigida Haenke
Gentiana froelichii Jan ex Rchb.
Gentiana futtereri Diels & Gilg
Gentiana × gaudiniana Thomas ex W.D.J.Koch
Gentiana gelida M.Bieb.
Gentiana gentilis Franch.
Gentiana gentilis subsp. eurycolpa (C.Marquand) Halda
Gentiana georgei Diels
Gentiana gilvostriata C.Marquand
Gentiana glauca Pall.
Gentiana grandiflora Laxm.
Gentiana × grandilacustris J.S.Pringle
Gentiana grata Harry Sm.
Gentiana grata var. bryophylla (Harry Sm.) Halda
Gentiana grata subsp. burmensis (C.Marquand) Halda
Gentiana × grisebachiana Rouy
Gentiana grumii Kusn.
Gentiana gyirongensis T.N.Ho
Gentiana × haengstii Hausm.
Gentiana handeliana Harry Sm.
Gentiana handeliana subsp. erectosepala (T.N.Ho) Halda
Gentiana haraldi-smithii Halda
Gentiana harrowiana Diels
Gentiana haynaldii Kanitz
Gentiana heleonastes Harry Sm.
Gentiana helophila Balf.f. & Forrest

Gentiana helophila subsp. dolichocalyx (T.N.Ho) Halda
Gentiana hesseliana Hosseus
Gentiana hexaphylla Maxim. ex Kusn.
Gentiana himalayensis T.N.Ho
Gentiana hirsuta Ma & E.W.Ma ex T.N.Ho
Gentiana hohoxiliensis S.K.Wu & R.F.Huang
Gentiana hooperi J.S.Pringle
Gentiana hugelii Griseb.
Gentiana huxleyi Kusn.
Gentiana × hybrida Schleich. ex DC.
Gentiana infelix C.B.Clarke
Gentiana intricata C.Marquand
Gentiana intricata var. subintricata (T.N.Ho) Halda
Gentiana jamesii Hemsl.
Gentiana jarmilae Halda
Gentiana jingdongensis T.N.Ho
Gentiana jouyana Hul
Gentiana kaohsiungensis Chih H.Chen & J.C.Wang
Gentiana kauffmanniana Regel & Schmalh.
Gentiana khammouanensis Hul
Gentiana kurroo Royle
Gentiana kurroo var. lowndesii (Blatt.) T.N.Ho
Gentiana kwangsiensis T.N. Ho
Gentiana lacerulata Harry Sm.
Gentiana laevigata M.Martens & Galeotti
Gentiana laevigata var. hintoniorum (B.L.Turner) T.N.Ho
Gentiana langbianensis A.Chev. ex S.Hul
Gentiana lateriflora Hemsl.
Gentiana lateriflora var. uncifolia (H.J.Lam) T.N.Ho
Gentiana latidens (House) J.S.Pringle & Weakley
Gentiana lawrencei Burkill
Gentiana laxiflora T.N.Ho
Gentiana leptoclada Balf.f. & Forrest
Gentiana leptoclada subsp. australis (Craib) Halda
Gentiana leroyana Hul
Gentiana leucomelaena Maxim.
Gentiana lhassica Burkill
Gentiana liangshanensis Z.Y.Zhu
Gentiana licentii Harry Sm.
Gentiana ligustica R.Vilm. & Chopinet
Gentiana linearis Froel.
Gentiana lineolata Franch.
Gentiana linoides Franch. ex Hemsl.
Gentiana loerzingii Ridl.
Gentiana longicollis G.L.Nesom
Gentiana loureiroi (G.Don) Griseb.

Gentiana loureiroi subsp. napulifera (Franch.) Halda
Gentiana lowryi Hul
Gentiana lutea L.
Gentiana lutea var. aurantiaca (M.Laínz) M.Laínz
Gentiana lutea subsp. montserratii (Vivant ex Greuter) Romo
Gentiana lutea subsp. symphyandra (Murb.) Hayek
Gentiana lycopodioides Stapf
Gentiana macgregoryi Hemsl.
Gentiana macgregoryi subsp. piundensis (P.Royen) Halda
Gentiana macrophylla Pall.
Gentiana macrophylla subsp. fetisowii (Regel & C.Winkl.) Halda
Gentiana macrophylla var. fetissowii (Regel & Winkl.) Ma & K.C. Hsia
Gentiana maeulchanensis Franch.
Gentiana maeulchanensis subsp. kunmingensis (S.W.Liu ex T.N.Ho) Halda
Gentiana mairei H.Lév.
Gentiana makinoi Kusn.
Gentiana manshurica Kitag.
Gentiana × marcailhouana Rouy
Gentiana × marceli-jouseaui Halda
Gentiana masonii T.N.Ho
Gentiana × media Arv.-Touv.
Gentiana meiantha (C.B.Clarke) Harry Sm.
Gentiana melandriifolia Franch. ex Hemsl.
Gentiana membranulifera T.N.Ho
Gentiana membranulifera var. intermedia (C.B.Clarke) T.N.Ho
Gentiana micans C.B.Clarke
Gentiana micantiformis Burkill
Gentiana microdonta Franch. ex Hemsl.
Gentiana microdonta subsp. omeiensis (T.N.Ho) Halda
Gentiana microdonta subsp. phyllopoda (H.Le$Av.) Halda
Gentiana microphyta Franch. ex Hemsl.
Gentiana mirandae Paray
Gentiana moniliformis C.Marquand
Gentiana muscicola C.Marquand
Gentiana muscicola subsp. lacinulata (T.N.Ho) Halda
Gentiana myrioclada Franch.
Gentiana namlaensis C.Marquand
Gentiana nanobella C.Marquand
Gentiana nerterifolia P.Royen
Gentiana newberryi A.Gray
Gentiana newberryi subsp. tiogana (A.Heller) Halda
Gentiana nipponica Maxim.
Gentiana nivalis L.
Gentiana nopscae (Jáv.) Wraber
Gentiana nubigena Edgew.
Gentiana nudicaulis Kurz

Gentiana nudicaulis subsp. lakshnakarae (Kerr) Halda
Gentiana nudicaulis subsp. ting-nung-hoae (Halda) T.N.Ho
Gentiana nyalamensis T.N.Ho
Gentiana obconica T.N.Ho
Gentiana occidentalis Jakow.
Gentiana officinalis Harry Sm.
Gentiana olgae Regel ex Schmalh.
Gentiana oligophylla Harry Sm.
Gentiana olivieri Griseb.
Gentiana oreodoxa Harry Sm.
Gentiana ornata (D.Don) Wall. ex Griseb.
Gentiana oschtenica Woronow
Gentiana otophora Franch. ex Hemsl.
Gentiana otophora subsp. sichitoensis (C.Marquand) Halda
Gentiana otophoroides Harry Sm.
Gentiana ovatiloba Kusn.
Gentiana ovatiloba subsp. chazaroi (H.H.Iltis) T.N.Ho
Gentiana pachyphylla Merr.
Gentiana × pallidocyanea J.S.Pringle
Gentiana pannonica Scop.
Gentiana panthaica Prain & Burkill
Gentiana panthaica var. epichysantha (Hand.-Mazz.) Harry Sm.
Gentiana papillosa Franch.
Gentiana paradoxa Albov
Gentiana parryae C.Marquand
Gentiana parryi Engelm.
Gentiana parvula Harry Sm.
Gentiana pedata Harry Sm.
Gentiana pedata subsp. xingrenensis (T.N.Ho) Halda
Gentiana pedicellata (D.Don) Wall.
Gentiana pedicellata subsp. glabriuscula (Harry Sm. ex T.N.Ho) Halda
Gentiana pedicellata subsp. zeylanica (Griseb.) Halda
Gentiana penetii (Litard. & Maire) Romo
Gentiana perpusilla Brandegee
Gentiana phyllocalyx C.B.Clarke
Gentiana piasezkii Maxim.
Gentiana piasezkii subsp. pubicaulis (Harry Sm.) Halda
Gentiana picta Franch. ex Hemsl.
Gentiana platypetala Griseb.
Gentiana plurisetosa C.T.Mason
Gentiana pneumonanthe L.
Gentiana praeclara C.Marquand
Gentiana praticola Franch.
Gentiana praticola subsp. greenwayae (Merr.) Halda
Gentiana prattii Kusn.
Gentiana primuliflora Franch.

Gentiana primuliflora subsp. melvillei (S.Moore) Halda
Gentiana producta T.N.Ho
Gentiana prolata Balf.f.
Gentiana prostrata Haenke
Gentiana prostrata var. crenulatotruncata C.Marquand
Gentiana prostrata var. karelinii (Griseb.) Kusn.
Gentiana prostrata var. ludlowii (C.Marquand) T.N.Ho
Gentiana prostrata subsp. nutans (Bunge) Halda
Gentiana prostrata var. pudica (Maxim.) Kusn.
Gentiana pseudosquarrosa Harry Sm.
Gentiana pseudosquarrosa subsp. ludingensis (T.N.Ho) Halda
Gentiana pterocalyx Franch.
Gentiana puberulenta J.S.Pringle
Gentiana pubigera C.Marquand
Gentiana pubigera var. glabrescens Harry Sm.
Gentiana pubigera subsp. ninglangensis (T.N.Ho) Halda
Gentiana pubigera subsp. pubiflora (T.N.Ho) Halda
Gentiana pulvinarum W.W.Sm.
Gentiana pulvinarum subsp. subtilis (Harry Sm.) T.N.Ho
Gentiana pumila Jacq.
Gentiana pumila subsp. delphinensis (Beauverd) P.Fourn.
Gentiana pumilio Standl. & Steyerm.
Gentiana punctata L.
Gentiana purpurea L.
Gentiana pyrenaica L.
Gentiana qiujiangensis T.N.Ho
Gentiana quadrifaria Blume
Gentiana radiata C.Marquand
Gentiana recurvata C.B.Clarke
Gentiana recurvata subsp. prainii (Burkill) Halda
Gentiana rhodantha Franch. ex Hemsl.
Gentiana riparia Kar. & Kir.
Gentiana riparia subsp. daochengensis (T.N.Ho) Halda
Gentiana robusta King ex Hook.f.
Gentiana rostanii Reut. ex Verlot
Gentiana rubicunda Franch.
Gentiana rubicunda subsp. biloba (T.N.Ho) Halda
Gentiana rubicunda subsp. delicata (Hance) Halda
Gentiana rubicunda subsp. filisepala (T.N.Ho) Halda
Gentiana rubicunda var. purpurata (Maxim. ex Kusn.) T.N.Ho
Gentiana rubicunda subsp. samolifolia (Franch.) Halda
Gentiana rubricaulis Schwein.
Gentiana sagarmathae Miyam. & H.Ohba
Gentiana saginifolia Wernham
Gentiana saginifolia var. montiswilhelmi (P.Royen) Halda
Gentiana saginoides Burkill

Gentiana saltuum C.Marquand
Gentiana saponaria L.
Gentiana satsunanensis T.Yamaz.
Gentiana scabra Bunge
Gentiana scabra var. buergeri (Miq.) Maxim. ex Franch. & Sav.
Gentiana scabra var. kitadakensis (N.Yonez.) Halda
Gentiana scabrida Hayata
Gentiana scabrida subsp. horaimontana (Masam.) Halda
Gentiana scabrida subsp. itzershanensis (T.S.Liu & C.C.Kuo) Halda
Gentiana scabrida subsp. luzoniensis (Merr.) Halda
Gentiana sceptrum Griseb.
Gentiana sedifolia Kunth
Gentiana septemfida Pall.
Gentiana septemfida subsp. grossheimii (Doluch.) Halda
Gentiana septemfida subsp. kolakovskyi (Doluch.) Halda
Gentiana septemfida subsp. overinii (Grossh.) Halda
Gentiana serra Franch.
Gentiana setigera A.Gray
Gentiana shaanxiensis T.N.Ho
Gentiana sierrae Briq.
Gentiana sikkimensis C.B.Clarke
Gentiana sikokiana Maxim.
Gentiana simulatrix C.Marquand
Gentiana sino-ornata Balf.f.
Gentiana siphonantha Maxim. ex Kusn.
Gentiana souliei Franch.
Gentiana spathacea Kunth
Gentiana spathulifolia Kusn.
Gentiana spathulifolia var. abaensis (T.N.Ho) Halda
Gentiana spathulisepala T.N.Ho & S.W.Liu
Gentiana squarrosa Ledeb.
Gentiana stellata Turrill
Gentiana stellulata Harry Sm.
Gentiana stipitata Edgew.
Gentiana stipitata subsp. tizuensis (Franch.) T.N.Ho
Gentiana stragulata Balf.f. & Forrest
Gentiana straminea Maxim.
Gentiana striata Maxim.
Gentiana stylophora C.B.Clarke
Gentiana suborbisepala C.Marquand
Gentiana subpolytrichoides Grubov
Gentiana subuliformis S.W.Liu
Gentiana sumatrana Ridl.
Gentiana susamyrensis Pachom.
Gentiana sutchuenensis Franch. ex Hemsl.
Gentiana szechenyii Kanitz

Gentiana taiwanica T.N.Ho
Gentiana takushii T.Yamaz.
Gentiana taliensis Balf.f. & Forrest
Gentiana tatsienensis Franch.
Gentiana terglouensis Hacq.
Gentiana terglouensis subsp. schleicheri (Vacc.) Tutin
Gentiana ternifolia Franch.
Gentiana tetraphylla Maxim. ex Kusn.
Gentiana tetrasepala Biswas
Gentiana tetrasticha C.Marquand
Gentiana thunbergii (G.Don) Griseb.
Gentiana thunbergii var. minor Maxim.
Gentiana tianschanica Rupr. ex Kusn.
Gentiana tibetica King ex Hook.f.
Gentiana timida Kerr
Gentiana tongolensis Franch.
Gentiana tonkinensis Hul
Gentiana trichotoma Kusn.
Gentiana trichotoma subsp. chingii (C.Marquand) Halda
Gentiana tricolor Diels & Gilg
Gentiana tricolor subsp. syringea (T.N.Ho) Halda
Gentiana triflora Pall.
Gentiana triflora subsp. japonica (Kusn.) Vorosch.
Gentiana tubiflora (G.Don) Griseb.
Gentiana uchiyamae Nakai
Gentiana ulmeri Merr.
Gentiana uniflora Georgi
Gentiana urnula Harry Sm.
Gentiana utriculosa L.
Gentiana vandellioides Hemsl.
Gentiana vandellioides subsp. *baoxingensis* (T.N.Ho) Halda
Gentiana vandellioides subsp. yiliangensis (T.N.Ho) Halda
Gentiana vandewateri Wernham
Gentiana veitchiorum Hemsl.
Gentiana venosa Hemsl.
Gentiana venusta (G.Don) Wall. ex Griseb.
Gentiana verna L.
Gentiana verna subsp. oschtenica (Kusn.) Halda
Gentiana verna subsp. pontica (Soltok.) Hayek
Gentiana verna subsp. tergestina (Beck) Hayek
Gentiana vernayi C.Marquand
Gentiana vernayi subsp. atropurpurea (T.N.Ho) Halda
Gentiana viatrix Harry Sm. ex C.Marquand
Gentiana villifera H.W.Li ex T.N.Ho
Gentiana villosa L.
Gentiana waltonii Burkill

Gentiana walujewii Regel & Schmalh.
Gentiana walujewii subsp. *tenuicaulis* (Ling) Halda
Gentiana wangchukii E.Aitken & D.G.Long
Gentiana wardii W.W.Sm.
Gentiana wardii subsp. *emergens* (C.Marquand) Halda
Gentiana wardii subsp. *micrantha* (C.Marquand) Halda
Gentiana wasenensis C.Marquand
Gentiana wilsonii C.Marquand
Gentiana wilsonii subsp. *striolata* (T.N.Ho) Halda
Gentiana winchuanensis T.N.Ho
Gentiana wingecarribiensis L.G.Adams
Gentiana wingecarribiensis var. *wissmannii* (J.B.Williams) Halda
Gentiana wootchuliana W.Paik
Gentiana xanthonannos Harry Sm.
Gentiana yakushimensis Makino
Gentiana yokusai Burkill
Gentiana yunnanensis Franch.
Gentiana yunnanensis subsp. *kialensis* (C.Marquand) Halda
Gentiana zekuensis T.N.Ho & S.W.Liu
Gentiana zollingeri Fawc.
Gentiana zollingeri subsp. *tentyoensis* (Masam.) Halda
